# Pathogenicity and virulence of *Neisseria gonorrhoeae*

**DOI:** 10.1080/21505594.2026.2749908

**Published:** 2026-10-16

**Authors:** Michael M. Girgis, Myron Christodoulides

**Affiliations:** a Neisseria Research Group, Molecular Microbiology, School of Clinical and Experimental Sciences, Faculty of Medicine, University of Southampton, Southampton, UK; b Department of Microbiology & Immunology, Faculty of Pharmacy, Mansoura University, Mansoura, Egypt

**Keywords:** Neisseria gonorrhoeae, gonorrhea, virulence, determinants, host response

## Abstract

*Neisseria gonorrhoeae* causes the sexually transmitted infection gonorrhea and is an obligate human pathogen, colonizing the mucosal epithelia of the male urethra and the female endo/ectocervix, resulting in urethritis and cervicitis. These manifestations represent uncomplicated gonorrhea and highlight the gonococcus as a primarily mucosal pathogen. However, gonococci can ascend the female reproductive tract to cause pelvic inflammatory disease and may disseminate to distant anatomical sites. The gonococcus expresses a diverse array of virulence factors that mediate transmission, mucosal colonization, immune modulation, and disease progression. In this review, we outline the mechanisms of gonococcal transmission and colonization of the genital tract epithelium, and examine host–pathogen interactions that drive inflammation, immune evasion, and immune suppression. We also discuss the mechanisms underlying dissemination and the resulting spectrum of clinical outcomes. Continued investigation of gonococcal biology and host interactions will advance understanding of pathogenesis and support the development of improved treatments and preventative vaccines.

## Introduction

*Neisseria gonorrhoeae* (Ng, gonococcus) is a Gram-negative Class β-Proteobacterium (Order Neisseriales, Family Neisseriaceae) and an obligate human pathogen. The gonococcus caused the sexually transmitted infection (STI) gonorrhea, and there was sufficient circumstantial evidence to show that this was a disease of antiquity and recorded clinically in many of humanity’s earliest surviving texts [1]. The organism was discovered by Albert Neisser in 1879 in methyl violet stained smears from 35 men and 9 women with purulent urethritis and two patients with acute ophthalmia [2], and the organism was subsequently named *Neisseria gonorrhoeae* in respect [3]. The early decades following the first isolation were characterized by the exhaustive demonstration of Koch’s postulates and the pathology of the disease from countless experimental gonococcal infection studies in humans [1]. The first drug to cure gonorrhea was sulfanilamide, introduced in 1938, and gonococcal resistance rapidly emerged to this drug and then to every other subsequent class of antibiotic ever developed and introduced. Nevertheless, antibiotic prescription remained highly effective, though the arsenal of antibiotics has dwindled as antimicrobial resistance (AMR) has increased inexorably. From 2024, third-generation cephalosporin-resistant and/or fluroquinolone-resistant gonococci now fall within the “High Group” of the World Health Organization (WHO) Bacterial Priority Pathogen List, and with Level 4 increasing trends in fluroquinolone resistant gonococci in two WHO regions [4]. During the latter half of the C20th, scientific studies have helped us to define the ultrastructure of the gonococcus, its virulence factors, the cellular immunobiology of gonococcal interactions with the host, and the nature of the host innate and adaptive immune responses. Moreover, the explosion of genome sequencing of the gonococcus − 30,000 genomes, and counting, in the PubMLST/*Neisseria* database – and the technologies that exploited these data, were now helping to develop effective treatments and potential prophylactics. In this review, we examined the pathogenesis of *N. gonorrhoeae* with a focus primarily on the molecular mechanisms of gonococcal virulence, and secondarily on treatments and vaccines. The authors were mindful of the fact that the gonococcal literature was enormous and that many detailed reviews on specific aspects of pathogenesis could be mentioned only cursorily herein.

### *The* N. gonorrhoeae *genome and proteome*

The reader is directed to the excellent review from Rotman and Siefert [5], which described the genome organization of the *Neisseriae*, the mechanisms of horizontal genetic exchange and the extensive phase and antigenic variation that occurred in the gonococcus that was fundamental to its pathogenesis. Phase variation was defined as the rapid and reversible switching of gene expression and was typically associated with genes encoding bacterial surface features [6]. Expression could be switched between ON and OFF states or between high and low levels of expression. This process could generate phenotypic diversity within a bacterial population, allowing subsets of bacteria to adapt rapidly to changing environmental conditions and evade host immune responses. Antigenic variation was defined as a mechanism by which bacteria altered the molecular structure or antigenic composition of their surface components, thus producing qualitatively different antigenic forms that could be distinguished by specific antibodies or antisera. Unlike phase variation, antigenic variation changed the antigen itself, which helped bacteria avoid recognition and elimination by the host immune system [7].

The dynamic processes of phase and antigenic variation allowed gonococci to rapidly switch on or off the expression of certain genes that enabled the bacteria to adapt to their changing environments and facilitate their survival. Briefly, the gonococcus contained an ∼2.2-Mbp circular chromosome, and using multiple methodological approaches, Tobiason and Seifert [8] showed that gonococci contained more than one complete genome copy per cell. Gene dosage analysis indicated that replication initiated only once per chromosome per cell cycle from a single origin of replication, located in a region not previously associated with canonical bacterial origins. Quantitative PCR demonstrated an average of three genome copies per coccal unit and overall, these findings supported a model in which at least two chromosomal copies were present per coccal unit and replicated synchronously to generate four chromosomes prior to cell division. Thus, gonococci were polyploid organisms, and the subsequent study from the same authors [9] further tested key predictions of this polyploidy model. Thus, gonococcal chromosomes were shown to exist as separate monomeric units rather than covalently linked dimers, consistent with the presence of distinct nucleoid regions observed by electron microscopy. Attempts to generate heterozygous cells, instead resulted in merodiploids, indicating that despite multiple genome copies, the bacterium remained genetically haploid. From these studies, polyploidy was proposed to facilitate antigenic variation, particularly the high-frequency gene conversion events underlying pilin variation, thereby promoting immune evasion and persistence within the human host.

There was also a significant amount and variety of repetitive DNA sequences in the genomes [10], of which the most abundant was the DNA uptake sequence (DUS) that mediated species-specific DNA transformation. The genomic flexibility of the pathogen was demonstrated by the presence of other repeats, e.g. the dRS3 (duplicated repeat sequence 3), Correia repeat, and the insertion sequences (IS)/minimal mobile elements (MME) [5]. The 20-base pair (bp) dRS3 sequence was the second most abundant repeat that could act as a site for phage integration. The 26-bp Correia repeat was present extensively in the gonococcal genome and often flanked a 100–150-bp DNA cassette as a terminal inverted repeat, known as a Correia repeat enclosed element (CREE) [5]. These CREE were important because they could fine-tune expression of virulence-associated genes, and they contributed to phenotypic variability between strains. This potentially aided adaptation to host niches via flexible transcriptional control and participated in regulatory network rewiring, including non-coding RNAs. In the gonococcus, IS were small mobile DNA elements (transposable elements part of the “mobilome”) that could move from one genomic location to another. Transposed within the genome, IS/MME could generate insertional mutations, rearrangements, and regulatory changes. Although they did not typically encode virulence or resistance genes themselves, their mobility contributed significantly to genome plasticity, gene regulation, and the evolutionary adaptability of gonococci [11].

There was also a genetic island (GGI), an ~57-kb DNA element present in 80% of gonococcal isolates that encoded a type IV secretion system (T4SS) that was required for extracellular secretion of single-stranded DNA for DNA transformation [12]. The gonococcus also contained bacteriophages in their genomes that contributed to genomic rearrangements [5]. There was also a stably maintained cryptic plasmid (4.2 kb pJD1) that was found in 96% of all gonococcal isolates [13]. Gonococci did not have endogenous Clustered Regularly Interspaced Short Palindromic Repeats (CRISPR) [14], but did possess several restriction-modification (R-M) systems to defend against the ingress of foreign DNA [15].

The first whole genome of *N. gonorrhoeae* sequenced was isolate FA1090, deposited in Genbank (accession No. NC_002946) in 2003 and in the PubMLST.org/*Neisseria* database [16]. The genome contained 2245 genes (total), 2174 Coding Sequences, and 1934 coding genes with protein (the proteome). With high-throughput genome sequencing, the PubMLST/*Neisseria* database now contained 36,506 gonococcal isolates and 29,939 individual gonococcal genomes and it was being continually updated. Another publicly available database that collected and analyzed gonococcal genomic data was curated by Pathogenwatch (https://pathogen.watch/), developed at the Big Data Institute and the University of Oxford. The availability of such large data could be used to quantify the gonococcal core genome and to understand the pathogen’s population biology, structure, and the emergence of AMR. In a study of 3750 gonococcal isolates collated from 1970–2018, a core genome of 1668 loci was identified [17] from the 2859 NEIS loci defined in PubMLST.org/*Neisseria*. There was also an associated chromosomal accessory pool of 247 genes that were located on large mobile genetic elements, which were associated with phage, or encoded putative secretion systems [18]. This accessory genome provided genetic flexibility to the gonococcus, which enabled it to adapt to its environment and host.

## The pathogenesis of gonococcal infection

Globally, ~87–100 million cases of gonococcal infection were reported annually, although this was likely an underestimate due to under-reporting of asymptomatic infection in both women and men [19–21]. Gonococci were not part of the normal microbiota of the urogenital mucosa, and primary colonization of the epithelium of the male urethra resulted in urethritis and painful discharge, and of the female endo/ectocervix, a mucopurulent cervicitis. In women, there was no vaginitis and the discharge was cervical in origin. Urethritis and cervicitis were presentations of uncomplicated gonorrhea and were characterized by infiltration of neutrophils. Thus, the gonococcus was a mucosal pathogen with a catalog of virulence factors that mediated the pathogenesis of infection and disease. Moreover, the gonococcus displayed both phase and antigenic variation, which will be discussed in context below. In this section, we follow the sequential pathogenesis of gonococcal infections, highlighting the molecular mechanisms and the nature of the gonococcal virulence factors involved. Pathogenesis involved bacterial transmission, the colonization of primary sites, dissemination to secondary sites, host innate immune responses and interrelated gonococcal-induced suppression of immunity, the development of clinical symptoms, and bacterial clearance.

### Transmission of gonococci and initial adherence to the lower reproductive tract cellular epithelium of the host

The gonococcus was principally a sexually transmitted pathogen, and men with asymptomatic infection comprised ~two-thirds of all infected men and were the major reservoir for spreading infection [22]. The gonococcus was transmitted mainly through unprotected vaginal, anal, or oral sex, and transmission rates from men to women were higher than from women to men [23]. Ejaculated seminal fluid contained millions of gonococci, and early studies demonstrated that gonococci were found attached to ~50% of ejaculated sperm. The primary adhesin used by the gonococcus to attach to sperm and other eukaryotic cells was a Type IV pilus (Tfp) [24]. It was also noted that the interaction between lipooligosaccharide (LOS) and the asialoglycoprotein receptor (ASGP-R) on human sperm served as a mechanism for gonococcal adherence and transmission [25]. This interaction may have facilitated the transmission of non-piliated as well as piliated gonococci. It has also been suggested that through sexual intercourse, a neutrophil-rich exudate could act as an efficient vehicle, because it harbored live and replicating bacteria, and stealthy transmitted the infection, much like a “Trojan” horse, to the other partner [21,26]. However, it was unclear how gonococci were transmitted from the vagina, rectum or oral cavity and pharynx to the male urethra, but the process probably depended on infected discharges and mechanical tissue damage. Existing evidence, albeit scant from a systematic review, suggested that tongue kissing may be a risk factor for oropharyngeal gonorrhea and gonococcal transmission, presumably via saliva, but not for *Chlamydia* [27,28]. Gonococcal infection also increased the risk of transmitting other pathogens, including *Chlamydia trachomatis, Treponema pallidum, Mycoplasma genitalium* and *Trichomonas vaginalis*. There was a strong association between gonococcal infection and human immunodeficiency virus (HIV) infection, where the gonococcus could enable HIV to cross the ano-rectal and genital epithelia, increase viral replication and shedding, and dysregulate the immune response to favor both pathogens. The interactions between gonococci and HIV were outside the scope of this review, and the reader was referred to excellent reviews elsewhere [29,30].

### The role of pilus

The primary adhesin used by the gonococcus to attach to eukaryotic cells was a Type IV pilus (Tfp). Other functions of the pilus included twitching motility by rapid extension and retraction [31] and the uptake of foreign DNA to increase transformation frequency [32]. Twitching motility was defined by Jorgen Henrichsen as “a kind of surface translocation produced by an unknown mechanism, which occurred in both flagellated and non-flagellated bacteria, but was never due to the action of flagella. The micromorphological pattern was varying but not as organized as in swarming and gliding. The cells moved predominantly singly, although smaller moving aggregates occur. The movement appeared as intermittent and jerky and did not regularly follow the long axis of the cell” [33,34]. Gonococci produced only Class I pili, and structurally, these Tfp were hair-like, flexible and helically homopolymeric fibers, 6 nm in diameter and several microns in length. PilE (the product of the *pilE* gene) was the pilin subunit that assembled into the multifunctional pilus adhesin and virulence factor: the crystallographic structure of the gonococcal pilin has been deduced and was reviewed in detail elsewhere [35]. When gonococci entered the lower genitourinary tract, they used type IV pili to establish initial contact with columnar non-ciliated mucosal epithelial cells of the urethra and ecto/endocervix, facilitating colonization of the host mucosa [36,37]. Gonococci also produced low amounts of a phase-variable PilC protein that was located at the pilus tip and involved in pilus-mediated adherence to ME-180 human uterine epidermoid carcinoma cells in vitro and to primary epithelial cells from the human cornea [38].

The first hypothesized eukaryotic cell membrane receptor for pilus was the membrane cofactor protein (MCP or CD46), which played a role in regulating complement activation [39]. Evidence to support CD46 as a pilus receptor included the ability of CD46 antibodies and soluble pilin to inhibit gonococcal attachment to ME-180 cells in vitro and to human tissues [40]. However, the role of CD46 has always been contentious and was largely disproven. For example, a Pil+ Opa- variant of *N. gonorrhoeae* strain FA1090 adhered with different efficiencies to several different human epithelial cell lines in vitro (Chang conjunctival, ME180, human endometrial adenocarcinoma (HEC-1B) and metastatic prostate adenocarcinoma (PC-3) [41]. There were four major isoforms of CD46, and these cells lines expressed different ratios of these isoforms, with no correlation found between CD46 isoform surface expression and pilus-mediated gonococcal adherence. This suggested that CD46 did not act as a classic receptor for gonococcal pili [41]. Also, there was evidence that pilus-mediated binding of gonococci to epithelial cells could occur independently of CD46; *in vitro* studies showed that 1) differences in binding efficiencies of piliated gonococci and purified pilus adhesin PilC2 on human epithelial cell lines (e.g. ME-180 and Chang conjunctival cells) did not correlate with the levels of surface-expressed CD46 [42]; 2) the lack of binding of both piliated gonococci and PilC2 protein onto CD46-transfected Chinese Hamster Ovary (CHO) and epithelial-like dog kidney (MDCK) cells; and 3) the fact that piliated gonococci or purified PilC2 protein bound equally well to normal epithelial cells and Hec1B and HeLa (Henrietta Lacks’s cancerous cervical tumor) cells with specific RNAi down-regulated CD46 expression [42].

Potential alternative receptors included 1) an uncharacterized surface protein that enabled pilus and the PilC2 pilus-associated adhesin to bind to human ME-180 epithelial and HBMEC (Human Brain Microvascular Endothelial Cell) lines in vitro [43]; 2) pilus binding to complement receptor 3 (CR3) that enabled adherence and invasion of primary human cervical epithelial cells in vitro [44]; and 3) pilus binding to I-domain-containing α(1)- and α(2)-integrins on primary male urethral epithelial cells and urethral tissue in vitro [45] (Figure 1). CD46 may well have bound pili, but these other receptors likely enabled early (i.e. <3 h) binding to urethral tissue and cells. In contrast, CD46 clustering and co-localization with gonococci were observed only at 6 h post-infection [44]. After pilus-mediated adherence, pilus retraction pulled the organism toward the membranes of primary cervical epithelial cells in vitro: this enabled the gonococcal porin and iC3b-LOS lipid A core complex also to bind to the I-domain and to serve as secondary adhesins [44,46].

**Figure 1.**
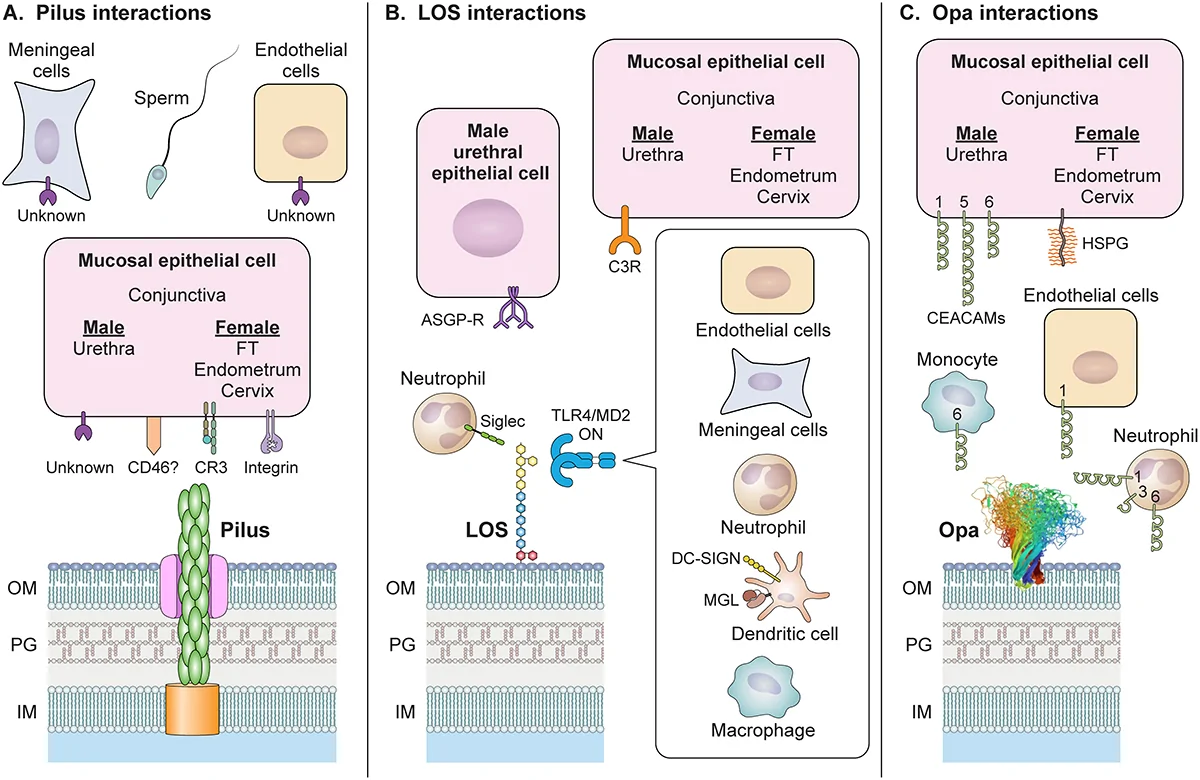
A cartoon showing the interactions of gonococcal pilus, LOS and opa with human cell receptors. See text for details. OM, outer membrane; PG, peptidoglycan; IM, inner membrane.

### Induced changes in host epithelial cells

What was clear was that gonococcal interactions altered host cell surfaces and modulated intracellular signaling pathways. Pilus attachment triggered a rapid and localized formation of cortical plaques in Chang, Hec-1B and A431 (human epidermoid carcinoma epithelial-like cells) in vitro, and these plaques contained f-actin and ezrin (components of the cortical cytoskeleton), and various integral membrane proteins, e.g. Heparan Sulphate ProteoGlycan (HSPG) CD44v3 that might bind Opa protein, the epidermal growth factor receptor (EGFR), and the CD44 and Intercellular Adhesion Molecule 1 (ICAM-1) adhesion molecules that mediated inflammatory responses [47]. Indeed, infection of HEC-1-B or ME-180 cells in vitro with gonococci upregulated ICAM-1 expression, which may have functioned to keep neutrophils at the site of infection, thereby reducing further invasion of the mucosa by gonococci [48]. Piliated, but not non-piliated gonococci that adhered to ME-180 cells in vitro, down-regulated CD46 surface expression by up to 80% [49], and this was accompanied by the specific shedding of this protein into the supernatant. When humans were experimentally infected with gonococci that could not induce CD46 down-regulation, variants of this strain were re-isolated that exhibited CD46 down-regulation [49]. Hypothetically, CD46 down-regulation could have represented a gonococcal-induced alteration in the host cell that promoted pathogen infectivity. Moreover, putative pilus binding to CD46 on A431 cells in vitro may not have been a static process, and CD46 may have promoted signaling cascades important for *Neisseria* infection [50].

CD46 existed as two major cytoplasmic isoforms, Cyt1 and Cyt2, which were generated by alternative splicing, and they differed in their intracellular tails, which conferred distinct signaling properties. Thus, Cyt2 contained Src-family kinase phosphorylation sites, whereas Cyt1 mediated alternative regulatory signaling pathways [50]. Gonococcal infection caused the aggregation of c-Yes, a member of the Src family of nonreceptor tyrosine kinases, in the eukaryotic cell cortex beneath adherent bacteria, increased the binding of c-Yes to CD46, and stimulated c-Yes kinase activity. This led to the rapid tyrosine phosphorylation of the tail of the Cyt2 isoform of CD46 with the involvement of c-Yes and recruitment of both Cyt1 and Cyt2 tails to the cortical plaques formed in infected human End1 endocervical and other epithelial cell lines in vitro [51]. Gonococcal infection also stimulated the processing of CD46-Cyt1 and CD46-Cyt2 in End1 cells in vitro by the Presenilin/γ-Secretase, which was an endogenous protease complex that regulated many important signaling proteins [52]. This stimulation was dependent on pilus and the ATPase PilT retraction motor. Moreover, proteolytic processing of CD46 that resulted in signal transduction via the Cyt1 and Cyt2 tails may have been important for regulating CD46-dependent cellular responses. Biologically, this cascade of events enabled downstream signaling pathways that regulated host epithelial cell responses, including adhesion stability, cytoskeletal organization, and immune modulation. Collectively, these processes reprogrammed host epithelial cells to promote gonococcal colonization and persistence.

After this initial attachment event, pili retracted very actively [53], and the generated force induced cytoskeletal arrangements in A431 epithelial cells in vitro [54]. Notably, pili formed bundles containing 8–10 pilus filaments, and this bundling process synchronized pilus retraction. The speed of retraction and twitching motility were enhanced by the action of PilT [55]. A consequence of motility and retraction was aggregation of gonococci into motile microcolonies that induced remodeling of the microvillus architecture and cortical actin plaque formation in A431 epithelial cells in vitro [56]. Oxygen was also required for the formation of these microcolonies by enhancing pilus-pilus interactions [57].

### Early gonococcal adhesion: a competitive interface

Transmission and initial adherence of gonococci to host epithelial cells constituted a dynamic competitive interaction, with both host and pathogen deploying mechanisms to establish dominance. One example of a host protective factor in non-pregnant women was a normal vaginal microbiota dominated by *Lactobacillus* spp., where the median pH was 3.9 in the lower vagina, 5.7 in the upper vagina, and not less than 7.7 in the upper uterine cavity [58]. This acidic vaginal environment was protective against gonococcal infection during transmission, whereas infection was significantly associated with a vaginal pH ≥ 4.5 and active menstruation [59]. In addition, other external stresses such as inhibitors of cell wall synthesis and DNA replication, antibiotics with different targets, the production of hydrogen peroxide and lactic acid, all inhibited pilus production [60]. These studies suggested that external stresses interfered with pilus functionality and would impact on gonococcal infectivity.

Semen also played a role within this competitive environment of transmission. Semen contained cationic amyloid fibrils, e.g. the semen-derived enhancer of viral infection (SEVI), which was reported to inhibit vaginal colonization of mice by gonococci [61]. Moreover, exposure to semen resulted in the dispersal of gonococci that had previously established surface adherence. Although this could be seen as host-protective by limiting colonization, it could conversely favor the gonococcus by enabling it to colonize other available epithelial cell surfaces.

Exfoliation and apoptosis of epithelial cells also were host defense mechanisms to counter colonization, but gonococci could contrarily use these processes for their benefit. For example, in experiments with human endocervical tissue explants in vitro, killing or shedding of epithelial cells was postulated to allow gonococci to penetrate deeper tissues and access protected niches, and thereby to promote bacterial survival and spread [62]. Additionally, the resulting cellular debris hypothetically overwhelmed or distracted phagocytes, and created temporary “immune blind spots” that gave gonococci the time to replicate before clearance [62,63]. Since genital tract mucosal epithelial cells produced key cytokines and chemokines that recruited immune cells, their loss would weaken local immune signaling and reduced the influx of neutrophils and other phagocytes [37].

The process of microcolony formation was another example of dynamic competition to establish dominance. Using cell-free culture conditions in vitro, the presence of *s*eminal plasma increased gonococcus-gonococcus interactions that contributed to the formation of these microcolonies and shear-resistant biofilms [64]. Following initial attachment to the host epithelium, gonococci aggregated into type IV pilus-dependent microcolonies [65–67] that remodeled the host epithelial cytoskeleton, e.g. in A431 cells in vitro [56], and altered intracellular signaling pathways. Microcolony formation on artificial surfaces and on three-dimensional human vaginal epithelial tissue in vitro was dependent on gonococcal expression of the extracellular DNA ligase LigE [68]. Microcolony formation appeared to be cyto-protective for the host cell epithelium because it reduced epithelial cell shedding, and consequently this allowed the gonococcus to establish infection. Microcolonies formed on the surface of HEC-1B cells in vitro, induced redistribution of host signaling molecules such as the EGFR and ErbB2 – a related molecule – which promoted epithelial responses that favored colonization and invasion [69]. In addition, activation of nuclear factor kappa-light-chain-enhancer of activated B cell (NF-κB) in infected human alveolar carcinoma A549 cells and ME-180 cells in vitro was temporarily associated with microcolony formation, and the potential activation of a protective inflammatory cytokine host response [70].

### Invasion of host epithelial cells by the gonococcus

#### The role of opacity (Opa) protein

The initial interactions mediated by pili retraction were crucial to overcome electrostatic repulsion and to position the gonococcus for more intimate interactions. The intimate and stable attachment of gonococci to host cells was primarily orchestrated by the hypervariable Opa (opacity-associated) proteins. These Opa proteins could be grouped into two major classes: 1) the Opa_HS_-type proteins (HS denoting the heparan sulfate-recognizing Opa proteins such as OpaA/Opa_30_ and Opa_50_); and 2) the majority Opa-CEA-type proteins such as OpaB-E, OpaG-K and Opa_CEA_ (the nomenclature defined originally from the MS11 isolate [71,72]) that bound tightly and specifically to members of the carcinoembryonic antigen-related cell adhesion molecule (CEACAM) family of receptors [73–75] (Figure 1).

Regarding the Opa_HS_ class of proteins, early studies showed that adherence of Pil-Opa+ gonococci to CHO cells in vitro was inhibited by heparin or heparan sulfate [76]. Subsequently, it was shown that the Opa_HS_ variants bound HSPGs, including the syndecan receptor proteins that were expressed on ME-180 cells and primary cultures of human corneal epithelium [77] and HeLa cells in vitro [78]. For the OpaA (Opa_HS_) variant, binding to serum vitronectin was necessary for invasion of CHO cells [79]. In addition, fibronectin was shown to bind to the Opa_50_ (Opa_HS_) variant, which triggered invasion of HEp-2 (laryngeal carcinoma epithelial cells) by co-ligation of HSPGs and the fibronectin-integrin receptor α5β1 [80]. The interactions between the majority Opa proteins and CEACAM receptors are described in detail below.

*Neisseria* Opa proteins contained eight antiparallel β strands that formed a barrel structure in the bacterial outer membrane (OM), which were linked by four extracellular loops [81]. Sequence variation was observed predominantly within extracellular loops 2 and 3, which were termed hypervariable HV1 and HV2, respectively. This sequence diversity defined Opa receptor specificity [82,83]. Opa proteins were originally named for their ability to confer an opaque morphology on gonococcal colonies under phase-contrast microscopy [84]. This opacity resulted from Opa-LOS interactions that occurred between the OMs of adjacent bacteria [85]. An important role for Opa during infection was suggested by the observations that Opa+ gonococci were recovered during natural gonococcal infection [86] and they were recovered also following inoculation of humans with Opa− bacteria [87,88]. Gonococci that differed in color and opacity, were distributed differently between male urethral and female cervical cultures. Colony characteristics of cultures isolated from female cervices differed with the use of oral contraceptives and the time of isolation within the menstrual cycle, with Opa− phase variants predominant during menses [86].

Recently, Opa variation was assessed quantitatively in a dataset of 219 complete genomes from phylogenetically diverse gonococcal isolates [89]. Each genome had on average seven distinct *opa* alleles at 9–12 *opa* loci, and almost all isolates had at least one pair of identical or near-identical *opa* genes. Among the 219 isolates, 193 (88%) had at least one *opa* gene in frame, and the mean number of *opa* genes in frame across all isolates was 1.7, and the maximum number of *opa* genes in frame in any isolate was five. The study revealed a continuously evolving *opa* repertoire. This expression heterogeneity enabled *N. gonorrhoeae* to adapt to different host environments and different cell types, thereby influencing tissue tropism and the outcome of infection [75,90,91].

### CEACAM-Opa interactions during adherence and invasion

The CEACAM family contained receptors that were expressed on a plethora of cells, including epithelial and endothelial cells, neutrophils, and lymphocytes [92,93]. Opa variations helped gonococci bind to different CEACAM receptors on these cells, thereby influencing innate and adaptive immune responses [83]. Epithelial cells displayed the pathogen-binding CEACAM1, 5, and 6, along with the non–pathogen-binding CEACAM7 [94,95] and neutrophils expressed CEACAM1, 3, and 6, as well as the non-binding CEACAM4 and 8 [96]. CEACAM1 and CEACAM3 were especially important, because both contained intracellular signaling domains that could trigger bacterial uptake, yet they propelled opposite cellular responses. CEACAM1 contained a long cytoplasmic tail harboring two immunoreceptor tyrosine-based inhibitory motifs (ITIMs). Following engagement by CEACAM-binding pathogens and phosphorylation of the ITIM tyrosine residues by Src-family kinases, CEACAM1 recruited the Src homology 2 (SH2) domain-containing phosphatases SHP-1 and SHP-2 [97–99]. These observations were made with human myelomonocytic JOSK-M cells [97] and primary human CD4+ T-cells in vitro [98] and with mouse T-cells in vitro, and with in vivo mouse models [99]. These phosphatases attenuated phosphotyrosine-dependent signaling by dephosphorylating key signaling intermediates involved in cellular activation, thereby suppressing pathways such as phosphatidylinositol-3 kinase (PI3K), mitogen-activated protein kinase (MAPK), and NF-κB signaling [97,99,100]. Consequently, the engagement of CEACAM1 dampened inflammatory responses, reduced cytokine production, and limited antimicrobial effector functions. In the context of gonococcal infection, CEACAM1-mediated signaling was proposed as an important immune evasion mechanism, enabling gonococci to exploit host inhibitory pathways to promote mucosal persistence and avoid effective innate immune clearance by neutrophils [100,101].

By contrast, CEACAM3 was a granulocyte-restricted receptor that contained a single immunoreceptor tyrosine-based activation motif (ITAM)-like sequence within its cytoplasmic domain. Following recognition of Opa-expressing gonococci by human primary granulocytes and isolated neutrophils in vitro, the tyrosine residues within the ITAM were phosphorylated by Src-family kinases, which created docking sites for the tandem SH2 domains of Syk kinase [102–104]. Subsequent Syk activation initiated an antimicrobial signaling network that involved phospholipase Cγ (PLCγ), PI3K, protein kinase C (PKC), Vav guanine nucleotide exchange factors, and the Rho-family GTPases Rac1 and Cdc42 [92,104,105]. These pathways drove extensive actin remodeling that facilitated rapid opsonin-independent phagocytosis of CEACAM-bound bacteria. Simultaneously, CEACAM3 signaling promoted NADPH oxidase assembly, reactive oxygen species (ROS) production, degranulation, chemokine secretion, and activation of NF-κB-dependent inflammatory programmes, which culminated in efficient bacterial killing by neutrophils in vitro [103,104]. CEACAM3 was therefore regarded as a dedicated innate immune receptor that linked pathogen recognition directly to antimicrobial effector functions.

The functional divergence between CEACAM1 and CEACAM3 represented an example of host–pathogen co-evolution. Whereas CEACAM1-mediated recruitment of SHP phosphatases established an inhibitory signaling environment that could be exploited by CEACAM-binding pathogens, CEACAM3 evolved as a specialized countermeasure against such microbial adaptation. Unlike CEACAM1, CEACAM3 converted bacterial adhesion into rapid phagocytic uptake, oxidative burst, and inflammatory activation, effectively restoring host defense mechanisms that would otherwise be suppressed through engagement of inhibitory CEACAM family members [103,104]. Comparative evolutionary analyses suggested that CEACAM3 emerged under strong selective pressure imposed by CEACAM-binding mucosal pathogens, including the gonococcus, leading to the development of a receptor uniquely adapted for the recognition and elimination of human-restricted bacterial pathogens [103,106]. Thus, Opa-expressing gonococci engaged CEACAM receptors, and differences in CEACAM expression on host cells may have influenced the fitness, selection, distribution, or recovery of Opa-expressing bacterial subpopulations [83,107–109]. These observations came from studies using Lec11 (a CHO derivative) cells in vitro [107], polarized human colon carcinoma epithelial-like T84 cells in vitro [109] and female transgenic mice expressing human CEACAMs [108].

For stable and local colonization, Opa could counteract host cell exfoliation, a defense mechanism that expelled gonococcal-infected cells. Using transgenic mice expressing CEACAM antigens [110] and ME-180 cells in vitro [111], this occurred through Opa-CEACAM interactions that induced expression of the matrix-binding protein CD105 (endoglin). In turn, this boosted integrin activation and the adhesion to extracellular matrix proteins, thereby creating a stable surface that facilitated mucosal colonization by the gonococcus. In studies with a human cervical tissue explant model, Yu et al. [112] demonstrated that gonococci colonized the ectocervix by activating integrin-β1, which inhibited epithelial shedding. Gonococci selectively penetrated the squamocolumnar junction (TZ) and endocervical epithelia by inducing β-catenin phosphorylation, which led to disassembly of the E-cadherin junction. As noted previously, epithelial cells in various cervical regions differentially expressed CEACAM molecules, with relatively high levels detected on the luminal membrane of ecto/endocervical epithelial cells, but very low levels intracellularly in TZ epithelial cells. The interactions between the gonococcal Opa_cea_ variant and CEACAM molecules increased colonization of the ecto/endocervix and reduced endocervical penetration by increasing integrin-β1 activation and inhibiting β-catenin phosphorylation, respectively. This occurred via CEACAM downstream signaling. In a different study, Opa and pili were involved in forming extremely tight and large microcolonies of gonococci that could hinder the ability of the bacteria to transmigrate across polarized T84 cells in vitro [113]. Taken together, both the intrinsic properties of cervical epithelial cells and phase variation of gonococcal surface molecules contributed to the regulation of gonococcal infection. This influenced gonococcal colonization of host epithelia, subsequent epithelial penetration, and, by implication, the development of either asymptomatic or symptomatic infection [112].

For intracellular invasion that followed the intimate association between the gonococcus and the host cell membrane, gonococci were enveloped within classical host cellular folds [36,114]. These interactions enabled gonococci to ‘’slip’’ into the sub-epithelial spaces or be taken up by complement-independent phagocytosis [115–117]. In epithelial cells, Opa-CEACAM interactions triggered signaling cascades described above, which led to actin rearrangement and microvilli elongation and engulfment. In addition, using Hec-1B and T84 cells in vitro, gonococci moved intracellularly from the apical to the basolateral side of the cell layer by activating EGFR, without necessarily breaching tight junctions [118]. This process allowed the bacteria to access sub-epithelial tissues and, in some cases, enter the bloodstream, leading to disseminated infection [119,120].

### The role of lipooligosaccharide (LOS) in adhesion and invasion

In addition to the pilus and Opa protein, LOS played a major role in gonococcal adhesion and invasion (Figure 1) [121]. LOS was a cardinal Pathogen Associated Molecular Pattern (PAMP) embedded in the OM and extending outwards, and it was composed of a lipid A, and an inner and outer core oligosaccharide. It was structurally distinct from the lipopolysaccharide (LPS) of Gram-negative enteric bacilli due to the lack of a repeating polysaccharide O-side chain [22,122]. The Lipid A component of the gonococcus was the primary site where the complement protein C3b was deposited [123]. This normally marked the bacteria for destruction by immune cells and helped activate the complement cascade to eliminate the pathogen. However, within the cervical epithelial cell microenvironment in vitro, this deposited C3b was rapidly converted into its inactive form, iC3b, on the bacterial surface [124]. Rather than promoting clearance, this modification benefitted the gonococcus. The iC3b coating, together with bacterial surface structures such as porins and pili, enabled the gonococcus to bind to complement receptor 3 (CR3) on cells of the female genital tract. This interaction enhanced bacterial adhesion and served as a key mechanism for inducing membrane ruffling and promoting invasion into cervical epithelial cells in vitro [44,46]. In addition, gonococci produced a phospholipase D enzyme that further promoted membrane ruffling and the clustering of CR3 receptors on the surface of primary cervical epithelial cells in vitro, which facilitated more efficient bacterial entry into host cells [125].

The interaction between LOS and the host ASGP-R found specifically on male urethral epithelial cells and sperm [25], triggered the formation of an actin-rich pedestal beneath the attached gonococcus [126,127]. It was also suggested that LOS was involved primarily in the rearrangement of actin filaments in gonococcal invasion of ME-180 cells in vitro, independent of Opa [128]. Once the pedestal was established, the ASGP-R facilitated endocytosis of the gonococcus through actin- and clathrin-dependent mechanisms, as demonstrated with Chang and T84 cells and with primary urethral epithelium in vitro [127,129].

### The involvement of nitric oxide (NO) during gonococcal infection

NO has also been implicated in gonococcal interactions, and it was produced by gonococci during low-oxygen (anaerobic) metabolism. NO activated a signaling pathway in human cells involving soluble guanylate cyclase (sGC), protein kinase G, and the transcription factor CREB (CAMP Response Element-Binding protein). This led to higher levels of CD105 expression, which made epithelial cells adhere more firmly to their substrate, and reduced shedding [130]. This was shown in vitro using 293T human embryonic kidney cells and in vivo with CEA-transgenic mice [130]. However, simply clustering CEACAM or binding it with inactive bacteria did not trigger increased CD105 expression, which demonstrated a requirement for live bacteria preconditioned by anaerobic conditions to make sufficient NO. Also, the close contact between gonococci and epithelial cells, mediated by Opa-CEACAM binding, was crucial to allow enough NO to reach the host cell cytosol [130], since NO functioned only over very short distances, despite being able to cross cell membranes easily [131]. This mechanism demonstrated that *N. gonorrhoeae* did not simply adhere to mucosal cells; it actively reprogrammed host-cell behavior through NO signaling to suppress epithelial exfoliation. By preventing the shedding of infected epithelial cells, the bacterium enhanced its ability to persist on mucosal surfaces, which promoted sustained colonization and potentially facilitated transmission and infection.

### Host cell autophagy and apoptosis

Once inside epithelial cells, gonococci could be challenged by intracellular autophagy and host cell apoptosis. Autophagy (from the Greek *auto* = self and *phagy* = eating) was a normal cellular housekeeping process in which cells captured and degraded their own damaged components, proteins, and invading microbes, and thus served as both a recycling system and an important antimicrobial defense mechanism [132]. Autophagy was activated early during invasion and could capture invading gonococci within the membrane-bound vacuoles of human urethral epithelial cells, which normally matured and fused with lysosomes to degrade the bacteria [126,127]. However, a subpopulation of gonococci could evade this fate by activating the autophagy repressor mTORC1 (mammalian target of rapamycin complex 1), which blocked autophagosome maturation and lysosome fusion in HeLa cells in vitro [133].

During early invasion of ME-180 and Hec-1B cells in vitro, gonococci rapidly recruited CD46-cyt1 to the cell cortex beneath the bacteria and triggered its cleavage by metalloproteinases and Presenilin/γSecretase. As infection progressed, gonococci gradually reduced CD46-cyt1 levels and remodeled lysosomes, which allowed the bacteria to avoid autophagy and survive within the host cell [134]. Gonococcal phospholipase A, a contact-dependent OM lysin, fragmented vacuole membranes by cleaving phosphatidylcholine, which helped the bacteria persist inside cells (both neutrophils and cervical epithelial cells in vitro) [135]. Additionally, gonococci secreted IgA protease, which cleaved the lysosomal membrane protein LAMP1 (lysosome/late endosome-associated membrane protein 1), which enhanced bacterial survival within the late endosomes and lysosomes of A431 cells [136] and bovine chromaffin cells [137] in vitro.

The other important defense mechanism that could prevent infection spreading was epithelial cell apoptosis [138,139]. Apoptosis was activated through two pathways: 1) the extrinsic pathway, which was triggered when death receptors on the cell surface were activated [140]; or 2) the intrinsic pathway, which was triggered by internal stresses such as ROS, or changes in cell pH that guided mitochondria to release the activating molecule cytochrome C [141–143]. Both routes activated a cascade of caspases (cysteinyl aspartate-specific proteinases), which resulted in host cell DNA breakdown, cell shrinkage, and membrane blebbing [144,145]. Bacterial infections often created intracellular stresses that triggered the intrinsic pathway, but *N. gonorrhoeae* was shown to protect human endocervical epithelial cells in vitro from apoptosis, probably as a mechanism that maintained an intracellular niche for the pathogen and thereby allowed it to evade immune clearance [146]. This mechanism was associated with a transient increase in the expression of the cellular inhibitor of apoptosis-2 (cIAP2) [146]. cIAP2 was also a potent inhibitor of apoptotic death in macrophages in vitro that was upregulated by LOS [147]. Furthermore, after invasion of human End/E6E7 endocervical epithelial cells in vitro, gonococci began to export exosomes that led to intracellular depletion of cIAP2, which initiated host cell death [148]. In addition, using HeLa2000 and Hec-1B cells in vitro, folliculin (FLCN), a 64 kDa protein with a tumor repressor function, was shown to be a novel host epithelial cell factor important for gonococcal survival after uptake, and this mechanism also involved modulating autophagy [149]. Recent studies in vitro with HeLa and HeLa ATG8 cells have also shown roles for the Nucleotide-binding oligomerization domain-containing protein 1 (NOD1) and immunity-related guanosine triphosphatase M (IRGM) protein in autophagy [150]. Pil-gonococci residing in early and late endosomes were detected and targeted by NOD1, which subsequently formed a complex with IRGM and the autophagy-related 16-like 1 (ATG16L1) protein. This activated autophagy and recruited microtubule-associated protein light chain 3 (LC3) to the intracellular bacteria. Furthermore, IRGM directly recruited syntaxin 17 (STX17) that could form tethering complexes with the lysosome [150].

Taken together, the central biological significance of these observations was that intracellular survival of *N. gonorrhoeae* within epithelial cells depended on a delicate balance between bacterial manipulation of host-cell death pathways and the host’s attempts to eliminate the pathogen through apoptosis and autophagy. The gonococcus promoted its intracellular survival by transiently suppressing epithelial-cell apoptosis to maintain a protected intracellular niche, while host cells attempted to eliminate invading bacteria through NOD1/IRGM-mediated autophagy. This highlighted the dynamic interplay between gonococcal immune-evasion mechanisms and host antimicrobial defenses.

### The role of gonococcal porin during infection: pro-apoptotic and anti-apoptotic effects

The gonococcal PorB.1A OM porin was associated with disseminated gonococcal infection (DGI) isolates, whereas the PorB.1B OM porin was more common to isolates causing localized genital infection [151]. PorB made up 60% of the total proteome of the gonococcal OM [152,153] and functioned as a voltage-gated pore, much like that of eukaryotic cell mitochondria. PorB mediated ion exchange between gonococci and its environment and was essential for bacterial viability, since it allowed acquisition of nutrients and modulated antibiotic diffusion into the cell periplasm [154,155]. For gonococci to enter epithelial cells, e.g. Chang conjunctival cells [156], they needed to form several tight contact points with the host epithelial cell membrane [157]. At these contact zones, PorB either attached to or integrated into the membrane, which triggered gonococcal uptake [156]. Integration of PorB into the host cytoplasmic membrane formed channels that could be tightly controlled by host cells. Patch-clamp experiments showed that PorB behaved like eukaryotic porins (voltage‑dependent anion channels , VDAC), with both bacterial and eukaryotic porins responding to voltage, as high voltage closed the channel. These specific features were mostly found with the PorB of pathogenic *Neisseria* species, and not in commensal species [154].

Once PorB translocated into the host cell cytoplasm of HeLa cells in vitro, for example, the evolutionary conserved mitochondrial import machinery could recognize PorB, as it shared several features with mitochondrial VDAC [158,159], which is involved in the regulation of apoptosis. The “translocase of bacterial origin” mitochondrial membrane proteins (the TOM complex, specifically TOM40 protein) recognized unfolded PorB and imported the protein into the mitochondria; however, PorB was not recognized by the sorting and assembly machinery (SAM) [160,161]. Once PorB was integrated into the mitochondrial membrane, it challenged the mitochondrial inner membrane (IM) potential and increased its permeability. The integrated PorB channel was found to be regulated by nucleotides. However, unlike other endogenous porins, mitochondrial integrated gonococcal PorB preferred an open state in the presence of ATP, even under high voltage conditions [154,160,162,163]. To further enhance intracellular survival within a variety of cell lines in vitro (HeLa, HEK293T and HCT116 human colorectal carcinoma), gonococci used PorB and OM vesicles (OMVs) to induce mitophagy (i.e. the removal of abnormal or dysfunctional mitochondria in epithelial cells), and to overcome the mitochondrial secretion of ROS [164].

There were strands of evidence that suggested that gonococcal porin interactions with host cells could be either pro-apoptotic or anti-apoptotic (Figure 2). In HeLa cells and phagocytes, porin translocation led to a rapid and transient increase in cytosolic calcium and subsequent activation of the Ca^2+^-dependent protease Calpain and caspases [165]. In macrophages, PorB interactions with host cell mitochondria led to the release of cytochrome C (CytC) into the cytoplasm, which triggered an intracellular caspase cascade (Caspase 3, −7) in a similar manner used by host cell mitochondria to induce time-dependent apoptosis [166]. In HeLa cells, the interactions of gonococci induced the release of the cytoskeletal proteins Bim and Bmf, which activated the downstream mutidomain effector pro-apoptotic proteins Bak and Bax; Bim itself was already a pro-apoptotic BH3-only protein [167]. These proteins perforated the outer mitochondrial membrane, and this was followed by the release of CytC. Subsequently, CytC bound to the apoptotic factor Apaf1 (apoptotic peptidase activating factor 1), which led to caspase activation [168]. Caspases then activated nuclear PARP (Poly (ADP-ribose) polymerase) that modulated DNA degradation and a pro-apoptotic phenotype. Conversely, purified PorB.1B was shown to upregulate the transcription of many host anti-apoptotic genes (e.g. *bfl-1, cox-2, c-IAP-2*) in human urethral epithelial cells in vitro, and transcription was dependent on NF-kB activation [169] (Figure 2). Porin was also reported to partially contribute to the anti-apoptotic effect in human endocervical epithelial cells infected in vitro with live gonococci [146].

**Figure 2.**
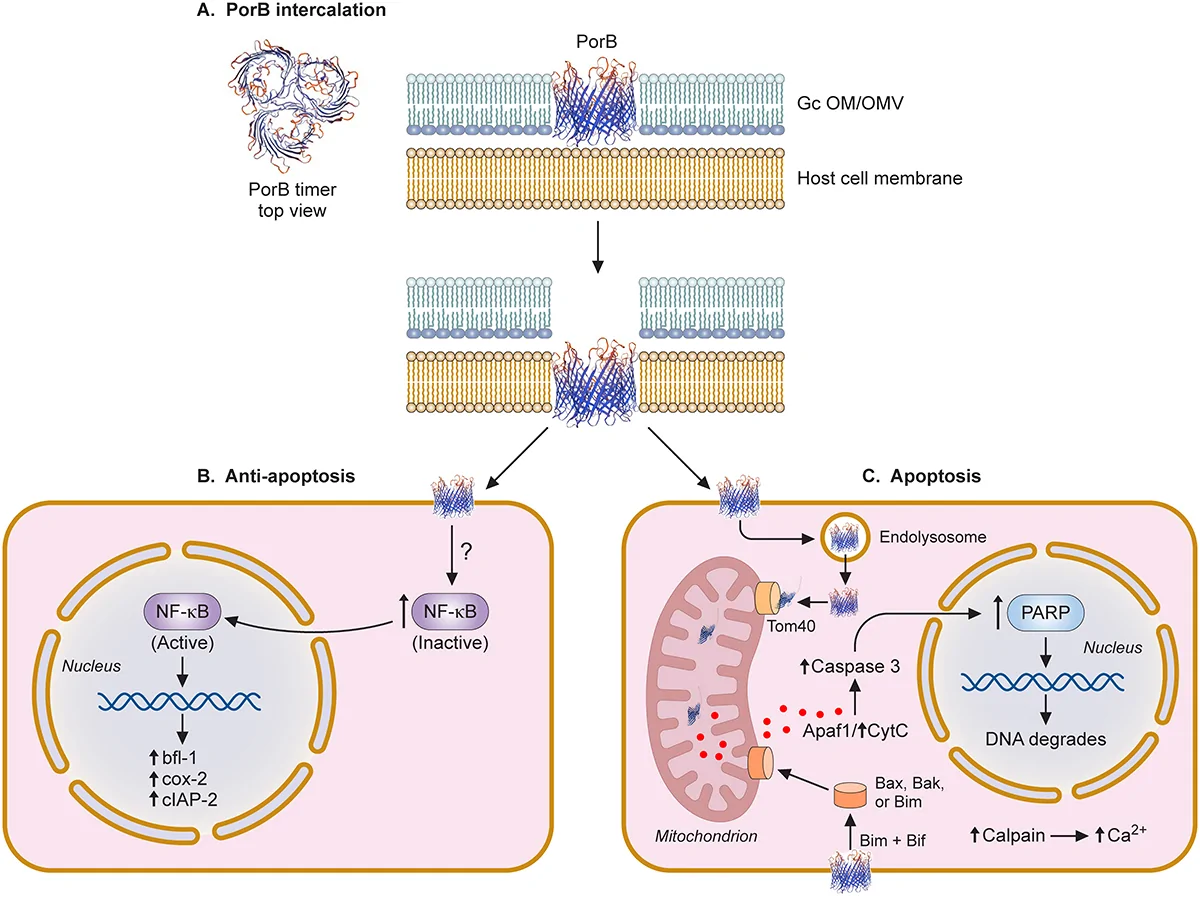
Interaction of *N. gonorrhoeae* porin B with human host cells. The structure of P20148 (OMPB2_NEIGO) *Neisseria gonorrhoeae* major outer membrane protein P.IB is shown (SWISS model). A) intercalation of porin into the host cell membrane; the structure of the porin consisting of three barrels was shown in side profile and a top view demonstrated the channel. B) anti-apoptosis in macrophages and human urethral epithelial cells induced by gonococcal porin. Purified PorB.1B upregulated the transcription of many host anti-apoptotic genes (e.g. *bfl-1, cox-2, c-IAP-2*) in vitro, and transcription was dependent on NF-kB activation. Porin was also reported to partially contribute to the anti-apoptotic effect in human endocervical epithelial cells infected with live gonococci. C) apoptosis: in HeLa cells and phagocytes, porin translocation led to a rapid and transient increase in cytosolic calcium and subsequent activation of the Ca^2+^-dependent protease calpain and caspases. In macrophages, PorB interactions with host cell mitochondria led to the release of cytochrome C (CytC) into the cytoplasm, which triggered an intercellular caspase cascade (caspase 3, −7) in a similar manner used by host cell mitochondria to induce time-dependent apoptosis. In HeLa cells, the interactions of gonococci induced the release of cytoskeletal proteins bim and bmf, which activated the pro-apoptotic proteins bak, Bax (bim itself was pro-apoptotic). These proteins perforated the outer mitochondrial membrane, and this was followed by the release of CytC. CytC bound to the apoptotic factor Apaf1 that led to caspase activation. Caspases then activated nuclear PARP that modulated DNA degradation and ended with a pro-apoptotic phenotype.

The modulation of host cell death by gonococcal porin was a mechanism that potentially contributed to the creation of a stable environment for gonococci to survive and replicate intracellularly. Thus, the observed pro- and anti-apoptotic effects of PorB were dependent on the cell-type, at least in vitro, and at different times during infection. A proposed interpretation was that early on, PorB.1B may have been anti-apoptotic when host cell nucleotide and nutrient levels were high, thereby enabling intracellular survival, whereas later, when the nutritional status was limiting, the pro-apoptotic phenotype would have enabled gonococcal dissemination and persistence of infection.

### Other virulence factors involved in adhesion and invasion

Besides the major roles of the pilus, Opa and LOS, a variety of other virulence factors contribute to gonococcal adhesin and invasion. These include the IgA1 protease, App and MspA/AusI autotransporters, OmpA protein, Maf proteins and lysozyme inhibitors (Figure 3).

**Figure 3.**
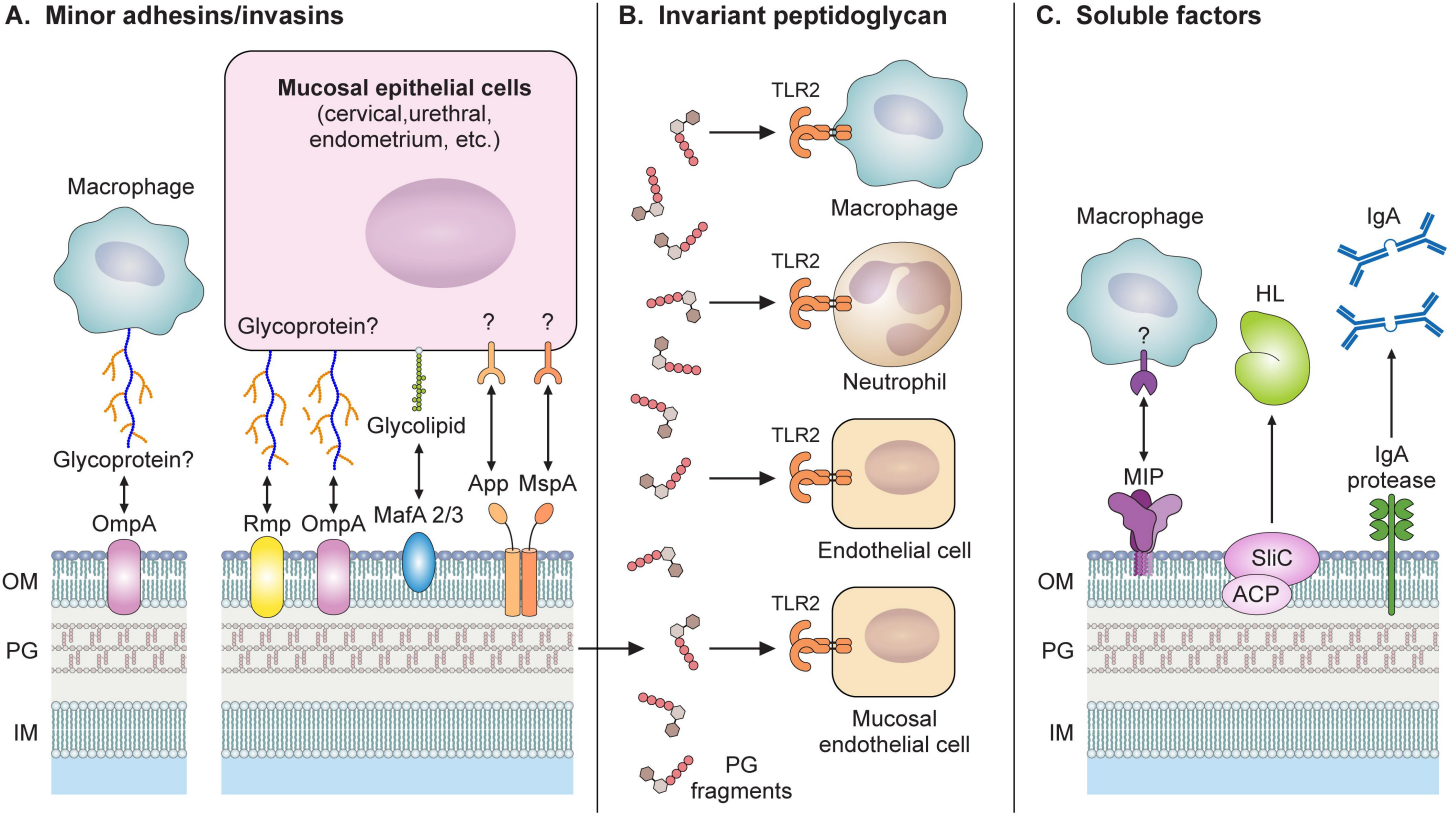
A cartoon showing the interactions of A) minor gonococcal adhesins/invasins and B) invariant peptidoglycan with human cell receptors and C) soluble factors (HL, human lysozyme, IgA antibody).

### The IgA1 protease

This was the first discovered classical autotransporter protein in *Neisseria* and its structure contained three parts – an N-terminal signal peptide, a secreted passenger domain, and a C-terminal translocator domain [170]. The N-terminal signal peptide could target the unfolded passenger domain, crossing the inner membrane to the periplasmic space via a secretion machinery. Next, the C-terminal translocator domain folded into β-barrel strands followed by insertion into the OM, a process that was facilitated by the Bam OM multi-protein complex machinery [171]. The gonococcal IgA protease was multifunctional, but essentially helped the bacterium to evade mucosal immunity and establish infection [172]. Primarily, it cleaved any human IgA1 antibodies present on genital tract mucosal surfaces, and thus the gonococcus avoided immune exclusion, antibody agglutination and Fc-mediated interactions with immune cells [173,174]. Consequently, the gonococcus could persist, despite any antibody response.

The IgA1 protease also indirectly supported colonization and invasion of mucosal urethral epithelial cells in the human challenge model, as cleavage of IgA bound to bacterial surface antigens may have reduced steric hindrance and improved interaction with epithelial receptors [175]. Johannsen et al [175] also demonstrated that an FA1090 *iga* mutant strain caused infection of male volunteers that was indistinguishable from the wildtype strain. Hedges et al. [176] demonstrated that there was no difference in the level of IgA1 protease activity in cervical fluid obtained from women who were, or were not, infected with *N. gonorrhoeae*. However, using a variety of cell lines (Chang, RT112 human bladder carcinoma, the human myelomonocytic cell line JOSK-M and myelocytic HL60 cells), Hauck and Meyer [177] reported that LAMP1 of epithelial origin was a target for IgA1 proteolytic cleavage. This was followed by additional studies in vitro from Ayala et al [178] and Lin et al [136] with A431 cells, which demonstrated that the IgA1 protease could cleave LAMP2, lysosomal acid phosphatase (LAP), and CD63, in addition to LAMP1. Although these data suggested a role for IgA protease in altering the lysosome, whether the enzyme contributed to gonococcal disease in men or women, in vivo, appeared unlikely [176]. Furthermore, gonococci did not need to produce the enzyme to colonize the male urethra and cause urethritis [175]. Moreover, although the IgA1 protease was immunogenic, it was hypervariable and antibodies against it would not reliably protect against reinfection [179].

### Autotransporters

The App (adhesion and penetration protein) and MspA/AusI (meningococcal serine protease A / autotransporter serine protease I) were two classical monomeric autotransporter adhesins that were secreted by *Neisseria* species via the type Va secretion pathway [180–182]. App adhesin was named after *Haemophilus* adhesion and penetration protein (Hap) of *H. influenzae* due to their strong homology [180]. App protein was secreted by all *Neisseria* species, and the amino acids sequences of the gonococcal App protein and meningococcal App showed 95% similarity [180,183]. The App passenger domain (of *H. influenzae*, as an exemplar) carried a self-associating region that formed intercellular multimeric complexes that could mediate bacterial cell–cell adhesion, aggregation, and biofilm formation [184]. The serine protease motif carried on the β-domain governed autoproteolytic cleavage and subsequent release of the passenger domain. Mutation in Ser267 abolished this autocatalytic activity, which confirmed the importance of the conserved catalytic triad (His115–Asp158–Ser267) [181].

App was a virulence-associated surface protein that contributed to adhesion, invasion, mucosal colonization, and immune modulation. The study by Xia et al reported that App was directly involved in mediating adhesion of gonococci to cervical epithelial cells in vitro, and antibodies against App inhibited this adhesion [185]. Evidence was also presented that App contributed to invasion of these cells, supporting a role for bacterial penetration into mucosal tissues. Moreover, the same study reported that fragments of App (passenger and translocator regions) induced strong IgG and IgA responses and a Th1 (IFN‑γ) and Th17 (IL‑17A) cytokine-driven immune response, which promoted opsonophagocytic and serum bactericidal killing of the gonococcus. Immunization studies showed that App-specific responses were associated with gonococcal clearance from the mouse vagina, suggesting biological relevance of its function in vivo [185].

MspA had some sequence homology to App (33%), IgA1 protease (36%), and other autotransporters, and it contained a catalytic triad (His100–Asp135–Ser241) within its passenger domain. MspA exhibited high genetic and phase variations and was expressed only within some *Neisseria meningitidis (*Nm, meningococcus) strains [182]. However, the *mspA* gene was absent in the gonococcal FA1090 isolate, and thus, the protein would play no role in pathogenesis.

### OmpA-like protein

The gonococcal OmpA-like protein was a highly conserved OM protein (OMP) that was related to OmpA proteins found ubiquitously in other Gram-negative bacteria [186]. The gonococcal OmpA-like protein, similarly to *E. coli* OmpA, played a significant role in the adhesion and invasion of human cervical carcinoma and endometrial cells in vitro and was needed for entry into macrophages and for intracellular survival [186]. In addition, gonococcal OmpA may have played an important role in colonization in vivo, since an *ompA* mutant demonstrated reduced recovery in the mouse model of infection, when compared with the wild-type strain [186].

Another major OMP of the gonococcus was the protein PIII (Rmp), which was 95% identical to RmpM (reduction modifiable protein M) or class 4 protein of *N. meningitidis*. The C-terminal domain of PIII (and RmpM) was highly homologous to members of the OmpA family. Purified PIII protein was shown to bind to human cervical and urethral cells in vitro and a *ΔpIII* knock-out mutant strain had a lower ability to adhere to the same cells [187]. These observations suggested a possible role of PIII in gonococcal virulence and that structural homology to OmpA was conserved at the functional level [187]. Recent studies have shown that *ompA* gene expression was subject to both iron-dependent (NceR) and -independent (MisR/MisS) pathways [188]. However, the receptor on the genital tract epithelium was not known, but very speculatively it may have been a cell surface glycoprotein related to heat shock proteins, in a similar manner as reported for the Omp-A protein of *E. coli* [189,190].

### Multiple adhesin family (Maf) proteins

Another family of proteins involved in adhesion were the Maf “Multiple Adhesin Family,” and this protein family was restricted to gonococci and meningococci. The *maf* genes represented 2% of the genome of these two species and encoded the MafA, MafB, and MafI proteins [191]. These proteins were all constituents of naturally secreted gonococcal OMVs [191–193]. MafA was an integral oligomeric OM lipoprotein of 312 amino acids and molecular weight of ~34 kDa that had both α-helical and β-sheet content [191,192,194]. These characteristics were like those found in CsgG, an OMP of *Enterobacteriaceae* involved in exporting Curli amyloids to the bacterial surface [195,196]. MafA was proposed to bind to glycolipid cell receptors on mammalian cells such as lactosylceramide, gangliotriosylceramide, and gangliotetraosylceramide [197,198]. A recent study showed that MafA 2/3 protein potentially played a role in gonococcal adhesion to Chang epithelial cells in vitro, independent of pilus and Opa expression [199].

The MafB protein was a secreted fratricide toxin with a globular structure, and it inhibited the growth of other bacteria by degrading RNA in a contact-inhibition manner [191,193]. It consisted of a N-terminal signal peptide, a conserved domain called DUF1020 and the highly variable C-terminal toxic domain. The latter domain could hypothetically allow the gonococcus to compete with the microbiome or pathogens that shared the same niches and typically lacked the ability to express MafI protein [191]. MafI was the complementary part of the toxin-immunity system that detoxified MafB toxins to protect *Neisseria* species from their own toxins [191,193,194]. This toxin-immunity system had no sequence similarities with TpsA (Two-partner secretion A) proteins, apart from the toxic domain [200], thus confirming the distinct evolution of the Maf secretion system by pathogenic *Neisseria* species. In pathogenic Gram-negative bacteria, the two-partner secretion (TPS) pathway, also known as Type Vb secretion, mediated the translocation across the OM of large effector proteins involved in pathogen-host cell interactions [201]. Briefly, a TPS system consisted of two proteins, the secreted TpsA effector protein and its TpsB partner transporter. The role of MafA protein in the toxin-immunity system was to mediate the transport of MafB across the neisserial OM. Once exposed on the neisserial surface, it interacted with an unknown receptor on the target cell, after which the C-terminal endonuclease domain was automatically cleaved and imported into the target cell, degrading its RNA. This secretion system was also suggested to contribute to the virulence of pathogenic *Neisseria* species by delivering MafB toxins into host cells [202].

Collectively, these observations suggested that the Maf system enhanced gonococcal colonization, persistence, and virulence by mediating epithelial-cell adhesion, eliminating competing bacteria through contact-dependent toxins [194], and potentially delivering effector molecules into host cells.

### Lysozyme inhibitors

The other protein family of note was the lysozyme inhibitors. Lysozymes were a component of the innate response to microbial colonization. During colonization, the gonococcus may have encountered c-type lysozyme in tears, saliva, urine, serum, vaginal fluid, cervical mucus, and in the lysosomal granules of neutrophils and macrophages [203]. The gonococcus had evolved sophisticated mechanisms to escape killing via chemical modifications of the peptidoglycan backbone [204] and by the synthesis of proteinaceous lysozyme inhibitors. The first lysozyme inhibitor was identified in *N. meningitidis*, and it was shown to function as an adhesin to human cells (Chang, Hep2, HUVEC (human umbilical vein endothelial cells), and meningioma) in vitro [205], and as a minor invasin, but no evidence of direct lysozyme inhibition was reported. Its role as a lysozyme inhibitor and its crystal structure were provided by Humbert et al [206], who reported that this meningococcal Adhesin Complex Protein (Nm-ACP) was structurally similar to the MliC/PliC (membrane-bound or periplasmic inhibitor of c-type lysozyme, respectively) protein family of lysozyme inhibitors. However, *Neisseria* ACP proteins showed < 20% primary sequence similarity with these MliC/PliC inhibitors and did not share any conserved MliC/PliC sequence motifs associated with lysozyme recognition [206]. *Neisseria* ACP probably adopted a different mode of lysozyme inhibition, and the ability of ACP to inhibit lysozyme activity was important for host colonization. The gonococcal ACP (Ng-ACP) was structurally and functionally homologous to Nm-ACP [207], and was localized in the OM/periplasmic inner leaflet and released extracellularly to some extent. Subsequently, a protein encoded by the open reading frame *ngo1063* was also shown to counteract c-type lysozyme and provide a competitive advantage in the murine model of gonorrhea. This protein was termed SliC (Surface-exposed Lysozyme Inhibitor of C-type lysozyme), but it also showed low overall primary sequence similarity to the MliC/PliC inhibitors, but a parallel inhibitory mechanism [208]. Indeed, it appeared that gonococci expressed dual lysozyme inhibitors, i.e. Ng-ACP and SliC (Ng_1063) [209]. Interestingly, mutation of Ng-ACP increased susceptibility to lysozyme, whereas the SliC mutant was only susceptible to lysozyme when Ng-ACP was inactivated [210]. Notably, lipidation of SliC was required for surface exposure of this inhibitor [210].

The biological significance of these observations was that *N. gonorrhoeae* possessed multiple mechanisms to resist one of the host’s most important innate antimicrobial defenses, namely lysozyme-mediated killing. Expression of the dual lysozyme inhibitors Ng-ACP and SliC enhanced bacterial survival on mucosal surfaces and within phagocytic cells by protecting the peptidoglycan layer from enzymatic degradation. The presence of two functionally overlapping inhibitors provided redundancy, ensuring effective protection against lysozyme encountered in secretions and within immune cells. Consequently, these inhibitors likely contributed to successful colonization, persistence, immune evasion and fitness during gonococcal infection.

### Other minor virulence factors

Other minor factors involved in adhesion and invasion were the products of the gonococcal min operon cell division genes, i.e. *minC, minD and oxyR*. Indirect evidence of their involvement was demonstrated by the reduced adherence to and invasion of human urethral epithelial cells in vitro as a consequence of defective microcolony formation in the mutant strains [211]. In addition, by recruiting host factor H, gonococci could co-opt CR3 expressed on primary human cervical epithelial cells. This represented a multifaceted virulence strategy that not only promoted complement evasion but also facilitated bacterial adherence and invasion of these cells in vitro [212].

## Host response to gonococcal infection

A cartoon showing how gonococci colonized the genital tract mucosae, moving from initial adherence to invasion, is shown in Figure 4. In this section, we describe the innate host response to gonococcal infection of the genital tract mucosae. Defense mechanisms employed by the host include nutritional immunity (NI), pathogen recognition, mucosal inflammation, and the interactions of a variety of immune effector cells.

**Figure 4.**
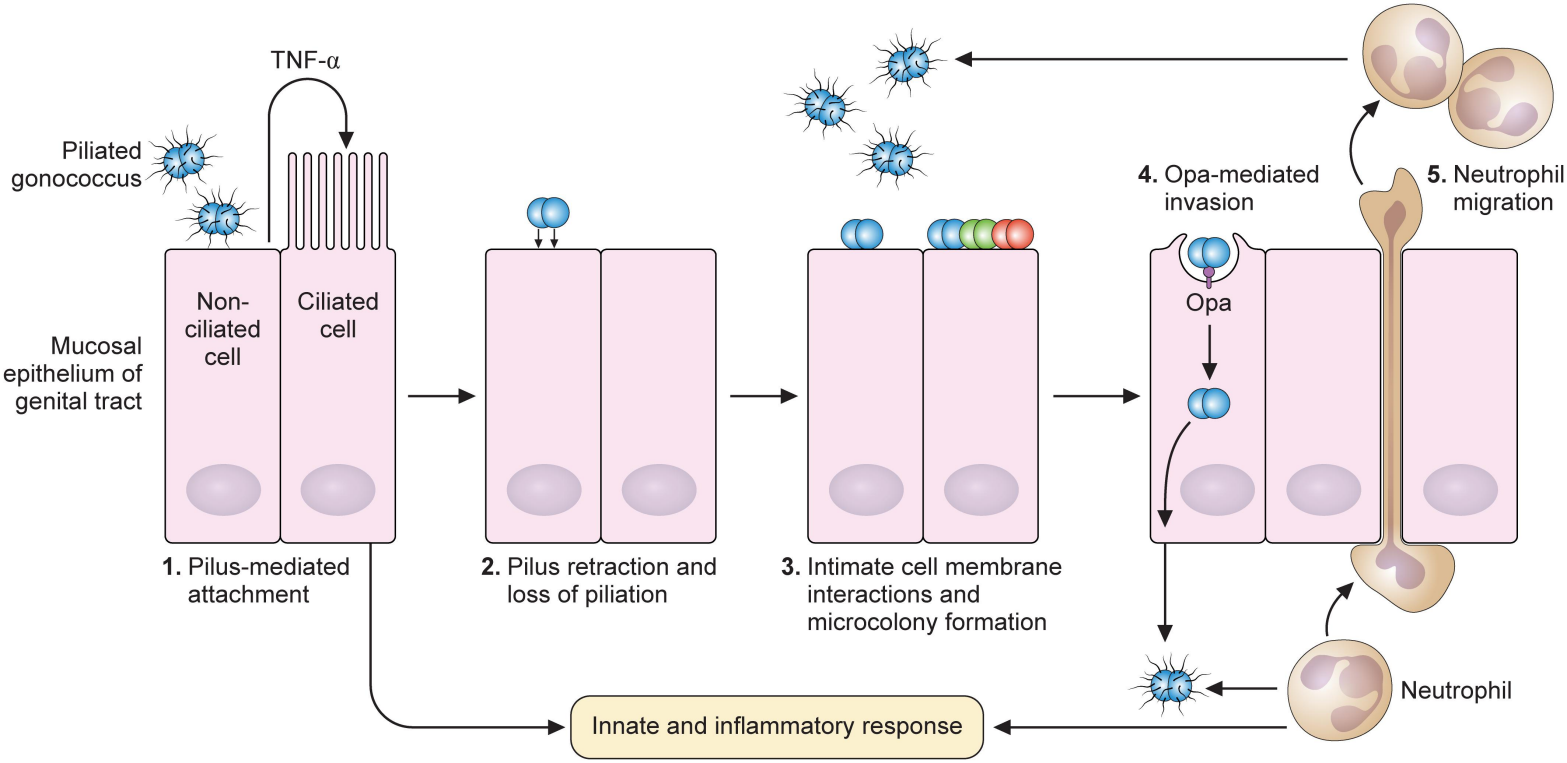
Model of the interactions between the gonococcus and host genital tract mucosal epithelial cells. 1. Piliated gonococci interacted initially with non-ciliated mucosal epithelial cells by binding to a putative receptor (whether it be CD46, an uncharacterized surface protein binding pilus and the PilC2 pilus-associated adhesin, the complement receptor 3 (CR3), or I-domain-containing α(1)- and α(2)-integrins); stimulation of TNFα release killed adjoining ciliated epithelial cells. 2. After initial binding, gonococci retracted their pili to move the bacteria closer to the cell surface. 3. The next stage was the beginning of intimate association between the gonococcus and the epithelial cell, which could also include bacterial division to form microcolonies. 4. Intimate interactions were mediated by Opa-CEACAM, and engulfment occurred through pedestal formation and invagination folds and host cell actin rearrangements. 5. Gonococci were now intracellular organisms that could pass through the cytoplasm to emerge into sub-epithelial spaces. 6. The host recognized the gonococcus pathogen associated molecular patterns (PAMPs) through pathogen recognition receptors (PRRs) such as TLRs and triggered intracellular signaling pathways to produce inflammatory signals. Neutrophils were recruited massively to the site of mucosal infection, and a pus discharge was generated.

### The concept of nutritional immunity

“Nutritional immunity” (NI) was a major defense mechanism used by the host against gonococcal infection. NI could restrict access to essential nutrients, in particular transition metals such as iron, zinc and manganese, and it was recognized as a fundamental arm of innate immunity. The term NI was first introduced by Eugene Weinberg in 1975 to describe the host’s attempt to withhold iron from microbial invaders (*sic*) [213]. NI was important because almost all bacterial pathogens, including the gonococcus, required metals for critical cellular processes. Thus, iron was required for DNA synthesis, respiration, and energy metabolism, zinc for enzyme function and protein synthesis, and manganese for protection against oxidative stress and enzyme activity [214]. When gonococci (or indeed other bacterial pathogens) entered the host, they encountered environments that were deliberately metal-poor, because free metals were virtually absent. The host sequestered metals using specialized proteins: for example, iron was sequestered by binding to transferrin, lactoferrin, ferritin, and heme-binding proteins, zinc by binding to calprotectin (S100A8/A9) and other S100 proteins, and manganese by binding calprotectin and related proteins. Briefly, in the case of iron, sequestration by the host has been described in detail by Moro [215] and involved 1) liver hepcidin-mediated systemic iron restriction; 2) neutrophil lipocalin-2 sequestration of bacterial siderophores; and 3) macrophage Nramp1 (Natural resistance-associated macrophage protein 1, SLC11A1)-mediated depletion of phagosomal metals. Calprotectin (CP) sequestered multiple divalent transition metal ions, including zinc and manganese [216]. CP was produced by epithelial cells at the site of infection and by invading neutrophils and within neutrophil extracellular traps (NETs), and withheld both metal ions from the gonococcus [217]. Thus, during the inflammatory process, described below, these mechanisms lowered metal ion availability at sites of infection, and this response could significantly limit bacterial replication. However, successful pathogens, including the gonococcus, have evolved mechanisms to overcome NI, and these mechanisms are described in more detail in the evasion section below.

### Pathogen recognition in gonorrhea

#### Toll-like receptor 4

The Pathogen Recognition Receptor (PRR) Toll-*like* Receptor (TLR)4 played a cardinal role in recognizing the PAMP LOS, which activated the production of pro-inflammatory cytokines and interferons (IFNs) that were responsible for innate and adaptive immune responses [121]. A protective role of TLR4 has been shown in experimental gonococcal infection of female mice [218]. The structure of the lipid A moiety of the gonococcal-*Neisseria* LOS was a critical determinant for TLR4 recognition. The lipid A moiety was a phospholipid composed of a 3-deoxy-d-mannooctulosonic acid (keto-deoxyoctulosonate [KDO]) and di-glucosamine unit with a high number of long-chain fatty acids. Lipid A was responsible for endotoxicity [219] and the immune-activating potential of LOS was influenced by the number of acyl (fatty acid) chains of the lipid A unit. The most potent forms of LOS typically had a lipid A structure with six (hexa-) acyl chains [220,221]. A reduced number of acyl chains negatively impacted biofilm formation and bacterial survival within male urethral epithelial cells in vitro [222]. It also modulated the activation of inflammatory signaling induced by TLR4 recognition of *Neisseria* LOS in a variety of cell types in vitro, including human dendritic cells (DCs), THP1 cells and mouse bone marrow derived macrophages [223–225]. The under-acylated lipid A, which was generated by natural mutations in the *lpxL1* gene responsible for adding secondary acyl chains to Lipid A in pathogenic *Neisseria* species, resulted in less invasive meningococcal infection in humans [226]. Data from the study of Zhou et al. using the mouse model of gonococcal infection [225] suggested that TLR4-mediated recognition of gonococcal LOS played an important role in the pathogenesis of gonorrhea, and that alterations in lipid A biosynthesis may have played a role in determining whether infections were symptomatic or asymptomatic.

Lipid A was not readily detected by the immune system when it was embedded within the OM. However, once LOS was freed from the bacterial surface, e.g. through OMV shedding or bacterial cell lysis, the lipid A portion could interact with TLR4-MD2 (myeloid differentiation protein 2) [227], which would lead to inflammation and toxic effects. The release of LOS as well as peptidoglycan – recognized by the PRR TLR2 (Figure 3)- and OMV in the sub-epithelial cells, activated extracellular TLR signaling and intracellular regulator signaling such as NOD1 in HeLa, HEK293, and AGS (human gastric adenocarcinoma) epithelial cells, macrophages, and DCs in vitro [228,229]. The NOD and TLR signaling from these cells activated inflammatory transcription factors and the release of cytokines and chemokines [229]. Human bone marrow derived macrophages and neutrophils exposed in vitro to LOS also produced type I IFN via TLR4/MD-2. Notably, IFNβ was detrimental to the host and favored gonococcal survival [230].

There were two other host detectors of LOS, additional to NOD and TLR signaling regulators, namely 1) a transient receptor potential (TRP) channel that detected LOS outside cells, particularly in the neuronal system causing neurogenic inflammation and pain [231] and 2) caspase-4/5 in humans (or caspase-11 in mice), which recognized LOS inside immune cells like macrophages to produce the pro-inflammatory cytokines IL-1β and IL-18, and an inflammatory cell death called pyroptosis [232,233].

Thus, the biological consequences of the recognition of gonococcal LOS by TLR4-MD2, NOD1, TRP channels, and cytosolic caspase-4/5, were the initiation of a complex host response characterized by pro-inflammatory cytokine and chemokine production, neutrophil recruitment, inflammasome activation, pyroptosis, pain signaling, and induction of adaptive immunity. This resulting inflammation contributed both to bacterial control and to tissue pathology.

#### ALPK1–TRAF-TIFA cytosolic surveillance pathway

In addition to classical pattern-recognition pathways like the TLRs, *N. gonorrhoeae* was likely sensed through the Alpha-Protein Kinase 1 (ALPK1)–TRAF (Tumor Necrosis Factor (TNF) Receptor-Associated Factor)-interacting protein with a forkhead-associated domain (TIFA) cytosolic surveillance pathway. Gonococci released heptose-1, 7-bisphosphate (HBP), a precursor of LOS, which activated this mechanism in 293T cells, Jurkat (immortalized human T lymphocyte) and THP-1 (a monocytic cell line derived from an acute monocytic leukemia patient) cells in vitro [234]. Thus, following delivery of bacterial heptose metabolites, ALPK1 phosphorylated TIFA, leading to TIFA oligomerisation, TRAF6 recruitment, and activation of NF-κB-dependent inflammatory responses. Local cytokine production was also observed in a mouse dorsal air pouch model after injection of HBP-containing supernatant [234]. This signaling cascade promoted the production of pro-inflammatory chemokines, including IL-8. Although the ALPK1–TIFA pathway has not yet been characterized extensively in *N. gonorrhoeae*, studies in other Gram-negative pathogens, including *N. meningitidis*, demonstrated that bacterial heptose metabolites activated ALPK1-TIFA signaling and drove NF-κB-mediated inflammatory responses. Consequently, this pathway represents a plausible mechanism contributing to the marked cytokine production, neutrophil recruitment, and tissue pathology associated with gonococcal infection [235–237].

#### cGAS-STING sensing pathway

Recent evidence also suggested that gonococcal infection might have activated the cytosolic DNA-sensing cyclic GMP–AMP synthase (cGAS)–STimulator Of INterferon Genes (STING) pathway alongside the TLR-, NOD- and TIFA-dependent signaling pathways [238,239]. Andrade et al. demonstrated in vitro using bone marrow derived macrophages and THP-1 cells, that gonococci activated this cGAS–STING pathway [230]. Whereas TLRs likely mediated the initial recognition of gonococcal surface components such as LOS, it was possible that cGAS–STING might have functioned as a “second-wave” intracellular danger-sensing pathway that was activated following gonococcal invasion, cellular stress, host cell damage, or the release of bacterial and host-derived DNA into the cytoplasm [240,241]. The activation of cGAS would generate the second messenger 2'3'-cGAMP, which would stimulate STING and downstream TBK1 (TANK-binding kinase 1) – IRF3 (Interferon Regulatory Factor 3) [242,243] and NF-κB signaling, leading to the production of type I interferons and pro-inflammatory cytokines [241,244].

In the genital mucosa, activation of cGAS–STING might therefore have contributed to the neutrophil-rich inflammatory response characteristic of gonorrhea. Importantly, while these responses amplified inflammation, they did not necessarily generate effective protective immunity. This conclusion was consistent with the ability of *N. gonorrhoeae* to induce robust innate immune activation while simultaneously limiting the development of durable adaptive immune responses and immunological memory.

#### Mucosal inflammation

Mucosal inflammation and cytokine production were observed in both urogenital [245] and rectal [246] mucosa of humans infected with gonococci. The cumulative data from in vivo and in vitro studies demonstrated the struggle between the host’s innate immune response to clear infection and the pathogen’s suppressive effects to establish infection (Figure 5). Experimental inoculation of the urethrae of human male volunteers to induce urethritis was accompanied by the rapid production of the inflammatory cytokines IL-1β, IL-6 and TNFα and the chemokine IL-8, detected in urine and serum, and an influx of leukocytes [247]. Cytokine production was detected in urine before the onset of symptoms, and IL-6 and IL-8 and possibly TNFα were produced at the local site of infection, whereas IL-1 β was derived from infiltrating leukocytes [245]. In addition, mucosal gonococcal disease has been shown to induce porin-specific circulating T lymphocytes of the Th2 phenotype, and a portion of these cells could potentially traffic to mucosal surfaces [248]. However, gonococci induced only a limited cytokine and antibody response during uncomplicated cervical infections, with levels of IL-1, IL-6, IL-8, IL-10, and TGFβ not elevated in genital secretions from infected women [249]. The differences in cytokine responses in men and women were also observed in a study of systemic and mucosal cytokines (IL-1β, IL-2, IL-4, IL-6, IL-10, IL-12p70, IL-17A, TNF-α, INF-γ) in gonococcal infection: in this study, 1) systemic IL-6, TNF-α and IL-10 levels were raised, while genital IL-10 and TNF-α levels were lower in gonococcal positive individuals; 2) the levels of genital IL-1β and IL-6 and systemic IL-6 were elevated in gonococcal-infected men; and 3) none of the genital cytokines were elevated in gonococcal-infected women, although all systemic cytokines, except INF-γ, were elevated in these infected women [250].

**Figure 5.**
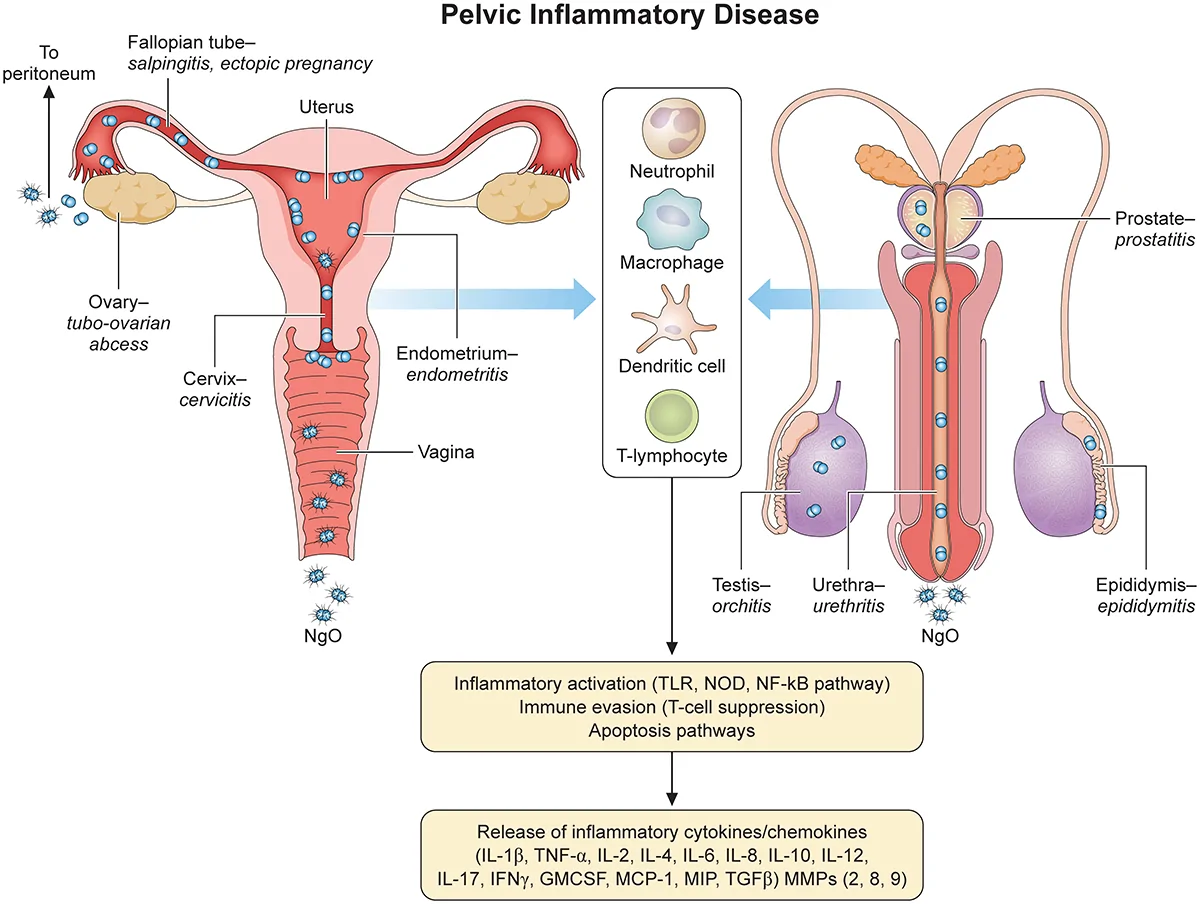
Gonorrhea is an inflammatory disease in both the male and female. In this cartoon, gonococci entering the female lower genital tract and the male urethra stimulated local inflammatory signaling pathways in the mucosae that led to the influx predominantly of neutrophils (a pus-forming response). In females, this was principally an ecto/endocervicitis (no vaginitis) and in the males, a urethritis. Several cytokines, chemokines and matrix metalloproteinases were measurable during infection; in the female, gonococci could ascend into the upper reproductive tract and induce pelvic inflammatory disease, which included salpingitis, endometritis, tubo-ovarian abscess formation, the risk of ectopic pregnancy, pelvic pain and escape of the pathogen into the peritoneum. In the male, gonococci could induce inflammatory responses within the testes, epididymis and prostate, and complicated infection could lead to infertility.

In vitro, gonococcal infection of HeLa, ME180 and HaCaT (immortal keratinocyte) cell lines induced the transcriptional factors NF-κB and Activator Protein-1 (AP-1), resulting in the expression of mRNA for GM-CSF, TNF-α, IL-6, IL-8, MCP-1, TGF-β, and IL-1β, and slight increases in IL-1α and IL-12 [251]. Furthermore, activation of NF-κB in A549 (lung carcinoma-derived) SIB01 epithelial cells in vitro was associated with microcolony formation by gonococci and the retraction of pili [70]. IL-6, IL-8 and IL-1 were also detected in epithelial cell lines derived from normal human endocervix, ectocervix, and vagina [252] and urethra [253], infected with gonococci in vitro. Early adhesion events were responsible for the induction of inflammatory responses in vitro [254]. However, gonococcal upregulation of Activating Transcription Factor 3 (ATF3), a transcription factor that negatively regulated the expression of many cytokine genes, in infected T84 epithelial cells in vitro, inhibited the expression of IL-6 [255]. Hypothetically, the early expression of IL-6 may have helped gonococci establish infection by preventing epithelial cell death and recruiting leukocytes to the infection sites, while later ATF3 expression prevented excessive inflammation that would have damaged both host and bacterium.

Virulence factors that induced cytokine release from eukaryotic cells in vitro included the classical PAMPs of LOS and peptidoglycan and its fragments, and the Lip lipoprotein, which induced TLR2-dependent release of IL-6 and IL-8 from infected End/E6E7, HEK 293, and HEK-TLR2 cells [256]. Somewhat counter-intuitively was the report that the lytic transglycosylases LtgA and LtgD, which served to release peptidoglycan fragments, reduced host innate immune signaling through TLR2 and NOD2 on THP-1 cells and DCs in vitro [257]. Thus, suppression of NOD2 activation by these enzymes may have provided a mechanism to suppress both innate and adaptive immune responses to the gonococcus, which enabled pathogen persistence and transmission. In support of this conclusion, recent data from using a human cervical tissue explant model in vitro, suggested that gonococci induced the local secretion of the anti-inflammatory cytokine IL-10 at the cervix, and they did this by engaging the host CEACAMs as a mechanism to prevent epithelial cells colonized by gonococci from being shed. It was suggested that this explained, in part, how gonococci colonized women asymptomatically [258].

Another aspect of the mucosal inflammatory response that favored the gonococcus, was the fact that epsilon-IFN produced in the female reproductive tract served to increase cytidine-5′-monophosphate (CMP)-sialic acid production in epithelial cells in the mouse genital tract, which in turn potentiated sialylation of LOS [259]. This latter effect was significant, since LOS sialylation in *N. gonorrhoeae* 1) enhanced bacterial survival by inhibiting complement activation (via factor H recruitment), 2) mimicked host surfaces, 3) suppressed innate immune responses (including neutrophil activity), and 4) increased resistance to antimicrobial peptides (AMPs). The cumulative outcome from LOS sialylation was colonization and persistence of the gonococcus [260].

Thus, the biological significance of this pro-inflammatory response was that it provided an essential mechanism for bacterial recognition, immune-cell recruitment, and infection control. However, the gonococcus subverted and modulated these same pathways to promote mucosal colonization, persistence, transmission and, in some cases, immunopathology. These are discussed further below.

#### Production of host protective factors

In addition to the pro-inflammatory response to gonococcal infection, the urogenital tract also produced antibacterial, suppressive and protective factors for host defense. This included lysozyme, AMPs and the presence of commensal organisms. Adhesion and invasion of human ME-180 epithelial cells in vitro by gonococci were inhibited by the presence of *Lactobacillus crispatus* and its enolase or glutamine synthetase enzymes [261]. Protection of ME-180 epithelial cells in vitro by the commensal was also extended to the reduction of TNFα and Chemokine (C-C motif) ligand 20 (CCL20) cytokine production during gonococcal infection [261]. Infection of HEC-1B epithelial cells in vitro also induced overexpression of long non-coding RNAs (lncRNAs), including MALAT1 (Metastasis Associated Lung Adenocarcinoma Transcript 1), ERICD (E2F1-Regulated Inhibitor Of Cell Death, Non-Protein Coding), and RP11-510N19.5 [262]. These lncRNAs regulated gene expression, including genes involved in immune signaling, and could have been involved in defense against infection. Production of the constitutive chemokine called monokine induced by gamma interferon (MIG)/Chemokine (C-X-C motif) ligand 9 (CXCL9) by male urogenital tract epithelial cells in vitro and its presence in seminal fluid, was shown to have anti-gonococcal activity [263]. Similarly, the CXC chemokine Granulocyte Chemotatic Protein-2 (GCP-2/CXCL6), which also had reported anti-gonococcal activity, was produced by epithelial cells of the male reproductive tract (as identified in formalin-fixed and paraffin-embedded samples of testis, epididymis, seminal vesicles and prostate) [264].

Thus, production of these host protective factors provided an important counterbalance to gonococcal virulence by reducing bacterial colonization, limiting inflammation and preserving mucosal integrity. The presence of commensal organisms, antimicrobial factors and host-derived chemokines could inhibit gonococcal adhesion, invasion, and survival, while regulatory molecules such as lncRNAs could strengthen local immune defenses. Collectively, these mechanisms contributed to natural resistance against infection and helped maintain urogenital tract homeostasis.

#### Gonococcal interactions with infiltrating neutrophils

Innate recognition of gonococci by the mucosal epithelium induced the inflammatory response, and this was accompanied by the ingress of neutrophils characteristic of gonorrhea [26]. The CEACAM3 receptor was expressed exclusively on human neutrophil surfaces, and the Opa-CEACAM3 interaction promoted neutrophil uptake of gonococci. In vivo, human CEACAM-expressing neutrophils had increased migration toward infection sites, where further activation occurred following gonococcal Opa-binding. Bacterial engulfment occurred via Src-family kinase-mediated trafficking of gonococci into mature phagolysosomes, and it initiated the oxidative burst and granule release required for pathogen destruction [93,265]. Engagement with CEACAM3 on neutrophils also triggered a Syk-, Protein kinase C-delta (PKCδ-), and Transforming growth factor-beta-activated kinase 1 (Tak1)-dependent signaling cascade that resulted in NF-κB activation and the production of more pro-inflammatory cytokines and chemokines [266] and leukotriene B4 (LTB4) [267]. Thus, this CEACAM3-Opa interaction would be expected to trigger mucopurulence with eventual pathogen clearance.

To avoid immune clearance, *N. gonorrhoeae* had evolved mechanisms to exploit neutrophils. Gonococci could survive, replicate, and persist within neutrophils, thereby gaining protection from extracellular immune effectors and creating an intracellular niche that favored persistence [268,269]. The intense neutrophil influx associated with symptomatic infection also generated metabolites such as lactate, which gonococci could utilize as alternative carbon and energy sources. Increased lactate availability enhanced gonococcal metabolism and oxygen consumption, which potentially impaired the oxygen-dependent antimicrobial mechanisms of neutrophils [270–272]. Furthermore, gonococci inhibited neutrophil apoptosis and suppressed ROS production through porin-mediated modulation of phagocyte oxidative metabolism, which prolonged neutrophil survival while reducing their bactericidal activity [273,274]. Collectively, these adaptations enabled the gonococcus to evade innate immune killing, to persist at mucosal sites, and enhanced its opportunities for transmission.

#### Gonococcal interactions with monocytes/macrophages

In the pathogenesis of gonorrhea, macrophages interacted with gonococci early in infection, but only after epithelial barrier engagement. Following mucosal colonization, gonococci penetrated or accessed the submucosa, where they encountered tissue-resident macrophages. During the acute phase, macrophages phagocytosed gonococci, yet the pathogen could evade killing, invaded macrophages, and even replicated intracellularly. Gonococcal interactions in vitro with Peripheral Blood Mononuclear Cells (PBMCs) were a pro-inflammatory event, with the generation of a wide variety of host signals. These included IL-8, the monocyte chemoattractant protein 2 (MCP-2), and a differential increase in the Th1 and Th2 associated cytokines IL-2, IL-4, IL-8, IL-10, IL-12, IFN-γ, TNF-α and MCP-1 [275]. LOS was probably the primary stimulant via TLR4. LOS was also implicated in activating the NOD-like receptor family pyrin domain-containing protein 3 (NLRP3)-inflammasome, which induced IL-1β secretion and pyronecrosis (cell death) of monocytes in vitro [276]. The biological significance of this pathway activation was a contribution to the inflammatory response and concomitant immune cell death, which might help to explain in part the contradictory host immune responses seen in natural gonococcal infection. In addition, Opa protein variation was reported to influence the production of pro-inflammatory cytokines from mature primary human macrophages in vitro [277]. The IgA protease was also reported to induce production by isolated PBMCs of TNF-α, IL-1β, IL-6, and IL-8 [278].

Gonococci could also invade macrophages, and they did this in groups as a colony of various sizes, by triggering actin skeleton rearrangement that caused the plasma membrane to form engulfing inward folds. The gonococcus-occupied vacuoles in the human monocytic cell line U937 escaped fusion with the endosomal compartment, surrounding itself within a network of actin filaments [279]. This invasion process was mechanistically distinct from phagocytosis that was regulated by the actin nucleating factor FMNL3 (Formin-like protein 3) and was independent of the Arp2/3 (Actin Related Protein 2/3) complex [279]. Gonococci survived within THP-1, U937, and primary monocytes, all differentiated into macrophages in vitro, for more than 24 hours by modulating apoptosis and cytokine production [280]. They may have polarized human macrophages in vitro to a M2 phenotype in a way that suppressed T cell proliferation by upregulating the immunosuppressive Programmed Death Ligand 1 (PD-L1) molecule [281]. Survival and replication within macrophages occurred regardless of nutrient availability and the immune activation status of the macrophage [282,283]. In fact, intracellular gonococci could re-program THP-1 and MM6 human macrophage-like monocytic cells in vitro to act as an “iron storage warehouse” by upregulating the expression of hepcidin, a master iron-regulating hormone, thereby increasing iron retention within the macrophage cytosol [282]. This mechanism was associated with downregulation of BDH2 (3-hydroxybutyrate dehydrogenase 2) expression, which mediated the synthesis of mammalian cell siderophore and acted in depleting freely available iron, thus contributing to host iron-limiting defense mechanisms [282]. In addition, the expression of a macrophage infectivity potentiator (MIP) protein promoted the intracellular survival of gonococci within RAW 264.7 cells (mouse monocyte macrophage) in vitro, suggesting a role for MIP in promoting the persistence of infection [284].

Collectively, these interactions highlighted a balance between host defense and immune subversion: macrophages contributed to pathogen recognition and inflammatory control of infection, whereas the gonococcus exploited macrophage survival pathways and intracellular resources to evade killing, to establish persistence and to promote ongoing transmission.

## Gonococcal evasion of host immunity

In this section, we described how gonococci employed multiple strategies to evade host immunity. These included evading NI, extensive gonococcal antigenic and phase variation including O-linked protein glycosylation, molecular mimicry, complement avoidance strategies, and active immune suppression.

### Avoiding nutritional immunity (NI)

As presented above, NI was a major host innate defense mechanism to limit gonococcal infection. Extensive discussion of how gonococci subvert NI was outside the scope of this review, and the reader was directed to a variety of excellent reviews elsewhere [285–287]. However, it did merit brief discussion herein. Most bacteria contested metal ion-specific NI by producing and secreting siderophores, which were low molecular weight iron-chelating compounds [288]. However, gonococci did not produce classical siderophores, but instead they absorbed metal ions directly from host-bound complexes and transported metals into the bacterial cell by using different means of transport complexes [286,289]. Thus, in response to iron-restrictive environments, gonococci produced Transferrin binding proteins (TbpA, B) [290], Lactoferrin binding proteins (LpbA, B) [291,292], and hemoglobin-binding OMPs (HpuA, HpuB) [293]. To overcome zinc and manganese restriction, gonococci expressed CbpA (calprotectin-binding protein A; formerly TdfH) [294], which was a TonB-dependent OM transporter protein that bound human host CP and extracted the metal ions from the host protein. This mechanism allowed gonococci to grow in zinc- and manganese-restricted environments and enhanced survival within neutrophil-rich inflammatory exudates and NETs [22,217].

An under-appreciated aspect of gonococcal pathogenesis was that during symptomatic gonorrhea, the intense neutrophilic inflammatory response paradoxically enhanced nutrient availability for the bacterium. However, most of the evidence was indirect and came from studies of neutrophil-rich inflammation, serum exudation, iron acquisition, and metabolic adaptation. The concept should not be overstated. The intense neutrophilic inflammation characteristic of symptomatic gonorrhea may inadvertently have increased nutrient availability through tissue damage, vascular leakage and the release of host-derived metabolites. Inflammatory exudates contained transferrin, lactoferrin, hemoglobin, and other potential nutrient sources that could be exploited by gonococci through their specialized uptake systems. In addition, neutrophil-derived lactate [272] was shown to enhance gonococcal metabolic activity, suggesting that inflammatory cell recruitment may have generated a metabolically favorable environment despite the antimicrobial functions of neutrophils.

In addition to immune pressures, metabolic factors could potentially shape gonococcal adaptation. One example of this was reported using a yeast two-hybrid system to identify human epithelial (HeLa) cell proteins that interacted with Opa proteins [295]. One of the proteins identified that co-localized with Opa was a pyruvate kinase (PK) isoenzyme M2. Using a gonococcal mutant that was unable to grow on pyruvate or lactate, it appeared that intracellular pyruvate was essential for gonococcal growth and survival. The authors suggested that this was a novel mechanism in gonococcal pathogenesis, i.e. by demonstrating a requirement for direct molecular interaction with a host metabolic enzyme (PK) for acquiring an essential intracellular carbon source and growth substrate (pyruvate) [295]. The advantage to the gonococcus was likely access to a reliable intracellular carbon and energy source within epithelial cells, which could enhance survival and persistence during infection. Interestingly, subsequent studies showed that PKM2 functioned not only as a glycolytic enzyme but also as an important regulator of immune-metabolic and inflammatory signaling pathways, including hypoxia-inducible factor (HIF)-1α-, Signal Transducer and Activator of Transcription (STAT)3-, and NF-κB-dependent responses [296]. However, no study has yet demonstrated that the gonococcal Opa–PK M2 interaction altered PKM2 signaling; thus, this remains an interesting hypothesis rather than a demonstrated mechanism.

### Examples of antigen and phase variation

Many gonococcal virulence factors were indispensable for pathogenesis, such that disruption of even a single gene could render strains noninfectious [297]. Importantly, gonococci did not simply express these factors constitutively; rather, their expression was tightly regulated and “fine-tuned” to optimize survival under specific host conditions. For example, infection of male volunteers with a *tbpB/tbpA* (transferrin-binding proteins) deletion mutant of strain FA1090 did not cause urethritis, and this was the first experimental demonstration in vivo that a bacterial iron acquisition system was an essential virulence factor for human infection [297]. As another example, modulation of the MlaA (the six-component maintenance of lipid asymmetry system) was critical for maintaining envelope integrity and appropriate virulence levels, as demonstrated in the mouse model of gonococcal infection [298]. Conversely, the MtrCDE efflux pump was not essential for establishing infection in the male urethra [299].

A particularly important adaptive mechanism involved the phase-variable Opa proteins, which enabled gonococci to respond dynamically to host environmental changes. In the female genital tract, a highly fluctuating environment shaped by hormonal cycles, Opa expression was influenced by anatomical site and endocrine status. Hormonal regulation altered host immune factors, including PRRs such as the TLRs [300], as well as AMPs [301] and lactoferrin production [302], mucosal pH, mucus viscosity, and microbiome composition [303]. These variations imposed selective pressures on gonococcal populations.

Experimental and in vivo studies showed that Opa expression contributed directly to fitness in this context. In female mice, Opa-expressing strains exhibited increased fitness over Opa-deficient mutants during early and late stages of infection. Correspondingly, Opa phase variation enabled gonococci to exploit or evade host factors throughout the reproductive cycle [304]. Clinically, Opa+ variants predominated prior to ovulation, whereas Opa− variants were more frequently isolated during menstruation, likely reflecting changing immune and biochemical conditions. For instance, complement component C3 expression was hormonally regulated, with distinct patterns between rodents and humans [305], while menstrual proteases such as trypsin preferentially selected for Opa− variants due to their increased resistance [86].

In contrast, in the male urethra, Opa+ variants were predominantly recovered during natural and experimental infections [86–88,306]. This selection coincided with the onset of inflammation, neutrophil recruitment, and exposure to serum components. *In vitro* studies further demonstrated that Opa+ variants exhibited greater resistance to serum-mediated killing than Opa− counterparts, which suggested that serum pressure contributed to the early transition toward Opa expression during infection [307].

### O-linked protein glycosylation

O-linked protein glycosylation played multiple roles for the gonococcus in interacting with the human host and avoiding innate immunity, and this was extensively reviewed by Borud and Koomey [308]. The *Neisseria* species contained numerous glycoproteins and displayed high intra- and inter-strain glycoform variability. The most notable of these was the *Neisseria* pilus, which showed a high degree of phase and antigenic variation [309] and post-translational modifications such as glycosylation [310–312], and phosphoform modifications (e.g. with phosphoethanolamine, phosphocholine, and phosphoglycerol) [313–317]. To date, over 50 glycoproteins have been identified in the gonococcus, and these were mainly lipoproteins and extracytoplasmic transmembrane–domain containing proteins [308]. Some of these glycoproteins were involved in AMR mechanisms (e.g. the MtrCDE efflux pump proteins, PorB/PenB, pilinQ and PonA) [318,319], some were considered as potential vaccine candidates, and some were involved in immune escape [308]. For the latter, glycan variation masked surface antigens and prevented effective antibody recognition, and several glycosylation genes (e.g. *pglA, pglE, pglG, pglH/H2, pglI*) contained repeat tracts that underwent slipped‑strand mispairing, which created high‑frequency ON/OFF switching [312,320–324]. The consequences of rapidly altering glycan length, composition, and O-acetylation status was intrastrain glycan heterogeneity, such that even a single isolate could express up to 15 different glycans [323,324]. The continuous generation of bacterial subpopulations pre‑adapted to immune pressure allowed the gonococcus to turn between glycoforms depending on the immune environment [308]. Horizontal gene transfer was also shown to expand glycan repertoires, and during colonization and invasive disease, gonococci accumulated glycosylation‑related mutations. Specific immune-modulating effects were also related to glycosylation, such as complement evasion and the example of pilin glycan contributing to the activation of CR3 to promote epithelial cell invasion. Collectively, these mechanisms undermined natural immunity [308].

### Gonococcal resistance to human complement

Key to gonococcal survival during infection was resistance to human complement. Complement proteins were ubiquitous and found in blood plasma, in interstitial fluids (tissues), on cell surfaces, at sites of infection or inflammation, and on microbial surfaces after activation [325]. LOS was the principal gonococcal determinant that was recognized by complement components present in human serum. Sialylation of gonococcal LOS converted complement serum-sensitivity to complement serum-resistance, but this depended on exogenously supplied sialic acid [326]. Sialylated gonococci inhibited 1) the classical complement pathway, by rendering the gonococci “self,” thereby reducing antibody recognition and binding [327], 2) the alternative complement pathway, through direct binding of sialic acid to factor H inhibitory molecule [328] or by enhancing the binding between the gonococcal PorB protein and factor-H inhibitory regulator [329,330], and 3) the lectin pathway, by reducing Mannose Binding Lectin (MBL) binding to LOS [331]. Sialylation also helped gonococci dampen neutrophil activity, independently of the complement system, via two mechanisms: 1) by reducing the ability of Opa-expressing gonococci to adhere to and stimulate neutrophils [332] or 2) by binding of sialic acid that “cap” the gonococci to Siglecs (sialic acid-binding immunoglobulin-type lectins) on the neutrophil surface [333].

Variations of LOS oligosaccharide core influenced favorably or adversely the sialylation process, spared the absence of other virulence factors and shaped the cytokine response. For example, gonococci expressing lacto-N-neotetraose were more invasive than gonococci that lacked this sugar molecule, since it was found to play a role in adhesion and invasion of ME-180 cells in vitro, in the absence of detectable Opa protein [128]. Sialylation of lacto-N-neotetraose, but not other sialic acid acceptors, was the only mechanism proven to enhance factor H binding to sialylated gonococci [334]. In shaping cytokine responses, the oligosaccharide core variations targeted different receptors on DCs, which modulated cell signaling to deviate the immune response from the production of complement-fixing antibodies. A shift toward a DC-induced Th-2 response might therefore have reduced the production of antibodies that activated the complement system, which could potentially slow down the host’s ability to clear gonococcal infections [335].

Even though LOS helped gonococci evade immune responses through antigenic variations, molecular mimicry, and immune suppression, anti-LOS antibodies were strongly associated with resistance against recurrent homologous infection of male volunteers [336]. Antibodies were believed to be transferred to the mucosal surfaces during the early stages of infection, and secretory IgA antibodies to LOS were found in all volunteers with gonococcal urethritis. IgG and IgM were also found, but in lower amounts. However, all antibodies (IgA, IgG, IgM) were depleted quickly in male volunteers infected with gonococci and the responses were short-lived [337,338].

Gonococci could make their own sialic acid, and during infection they sialylated the tips of their LOS by scavenging the small amount of cytidine-5′-monophosphate-N-acetylneuraminic acid (CMP-NANA; the donor molecule for sialic acid) present in the human or murine host [339,340]. From mouse studies, the main purpose of sialylating gonococci was to keep the bacteria in “silent” mode to persist as long as possible inside the host [340,341]. Sialylated gonococci were generally less invasive in challenged human male volunteers and tended to induce asymptomatic infection [342]. Thus, desialylation of LOS helped gonococci invade non-ciliated epithelial cells of the cervix of women and the urethra in men. This process was facilitated by the interaction between the desialylated LOS and the ASGP-R receptors, as shown on HepG2 (human liver cancer) cells in vitro [37,343]. The sialylation process was catalyzed by the LOS sialyltransferase (Lst) enzyme present on the gonococcal OM surface [344], and gonococcal strains expressed different levels of Lst [345]. Under laboratory anaerobic conditions, Lst boosted its activity four-fold, doubling sialylation speed and incorporating six times as much sialic acid onto gonococcal LOS [346]. This was relevant to the mêlée between gonococci, normal flora, and immune cells (particularly neutrophils) that needed a continuous oxygen supply to produce their reactive oxygen intermediates [347]. The presence of lactate or pyruvate during early bacterial growth boosted Lst enzyme activities, which sped up metabolism, growth, and protein production [348–350]. Boosting Lst activities enhanced the sialylation of gonococcal LOS, which promoted resistance to killing either by the complement system or by phagocytosis [351,352].

The cervicovaginal microbiome also played a key role in regulating sialylation, as it pumped high levels of sialidase enzyme into cervicovaginal secretions, which were capable of desialylating LOS. Although this made the gonococcus sensitive to complement, desialylation facilitated receptor-specific epithelial cell invasion during transmission from women to men. This transmission between genders was also dependent on other bacteria [353]. In this study of women with gonococcal infection, sialidases produced by the cervicovaginal microbiome bacteria *Gardnerella vaginalis, Prevotella species, Atopobium vaginae* and *Mobiluncus species* were suggested to increase the infectivity of gonococci during sexual transmission to men [353].

Gonococcal PorB protein also played a cardinal role in serum complement resistance. This protein could inhibit 1) the alternative pathway, through binding to factor H inhibitory regulator, and 2) the classical and lectin pathways, through binding to C4b binding-protein (C4BP). C4BP was a potent inhibitory regulator and acted as a cofactor for FI cleavage of C4b [354]. Sialylation of LOS was essential to enhance PorB-factor H binding to initiate serum complement resistance [330]. However, PorB-factor H binding could occur regardless of LOS sialylation [355]. In contrast, sialylation of LOS enhanced the binding between PorB and complement factor H-related protein 5 (CFHR5). PorB was unable to distinguish factor H inhibitor from CFHR5 that in turn, much like an “Achilles” heel,’ induced complement-mediated lysis of the bacterium [356]. Since PorB was invariantly expressed, PorB-factor H binding provided the gonococcus with a backup mechanism to reduce complement activation; this occurred when LOS could not be sialylated due to phase variation or lack of sialic acid [153]. PorB.1A and PorB.1B also showed differences in their capacity to bind C4BP: 90% of clinical isolates expressing PorB.1A were able to bind C4BP, whereas only 19% of strains expressing PorB.1B demonstrated this ability [357].

Rmp protein, which maintained the trimeric structure of PorB, helped to maintain the complement resistance function of PorB by eliciting “blocking antibodies” [358]. These were antibodies that bound to antigen with no action, and interfered with the ability of bactericidal antibodies to bind to the bacterial surface, thus preventing them from initiating complement-mediated killing [359]. This finding may explain why bactericidal activity in convalescent serum from patients with previous DGI was usually very low, regardless of high antibody levels [358]. Furthermore, a gonococcal Rmp deletion mutant induced antibodies with higher bactericidal activity than the parent strain [360]. In a classical long-term study involving 243 female sex workers in Nairobi, Kenya, women with circulating anti-Rmp antibodies were more susceptible to re-infection [361]. Moreover, anti-Rmp antibodies generated in the mouse model of infection appeared to reduce the binding of C3 complement molecules to the bacteria and increased the burden and duration of infection [362]. Thus, Rmp provided a key example of immune evasion by *N. gonorrhoeae* and demonstrated that some antibody responses were detrimental rather than protective. This discovery explained, in part, why natural infection failed to generate reliable immunity, and informed vaccine design by identifying Rmp as an antigen to avoid.

### Gonococcal modulation of the T-cell response

Th1 and Th2 cells were two major subsets of CD4^+^ T-helper cells that coordinated different types of adaptive immune responses. Th1 cells were most important for defense against intracellular pathogens and acted primarily by activating macrophages and cytotoxic T cells (cell-mediated immunity). Th2 responses were most important against extracellular pathogens and stimulated B cells and antibody production (humoral immunity) [363]. However, gonococci have evolved mechanisms that actively diverted the host adaptive immune response away from protective immunity and toward a predominantly Th17-driven inflammatory response. Studies in mouse models demonstrated that gonococci suppressed the development of Th1 and Th2 responses, largely through TGF-β-dependent mechanisms induced by gonococcal components such as Opa proteins [364]. Concurrently, gonococci promoted Th17 differentiation through the induction of TGF-β and activation of TLR4 signaling by LOS [364]. They also inhibited the DC-induced proliferation of human T-cells [365].

The resulting Th17-skewed response was characterized by enhanced production of IL-17 and the recruitment of large numbers of neutrophils to the site of infection. This response contributed to the pronounced neutrophilic inflammation that was a hallmark of gonorrhea. In murine genital tract infection, Th17-driven responses promoted substantial neutrophil influx and activation of innate defense mechanisms [366]. However, despite generating robust inflammation, these responses were poorly protective, and they simultaneously suppressed the development of Th1- and Th2-mediated immunity, thereby limiting the generation of effective immune memory.

Evidence from human infection supported this model. Acute gonococcal infection was associated with transient reductions in circulating CD4^+^ and CD8^+^ T-cell populations, with responses recovering following bacterial clearance [364]. Cytokine profiles in human infection were more heterogeneous than those observed in experimental models, with some studies reporting induction of IL-17A and IL-23, consistent with Th17 activation [367]. By contrast, other studies have documented variable IL-17 responses, suggesting site-specific differences in immune activation [368]. Nevertheless, inflammatory cytokines and neutrophil-recruiting mediators were consistently prominent, while Th1-associated cytokines, such as IFN-γ, remained relatively weak, unless experimentally enhanced.

Collectively, these findings suggested that the gonococcus exploited host immune pathways to favor inflammation rather than protection. The Th17-biased response functioned as an immune diversion strategy, generating an inflammatory environment that may have aided bacterial persistence while impairing the development of effective Th1- and Th2-dependent immunity. Consequently, antibody responses following infection were generally low, short-lived, and poorly protective, and immunological memory was weak or absent [369]. This represented a highly adapted host–pathogen interaction in which immune activation was effectively co-opted to benefit the pathogen rather than the host.

### Gonococcal suppression of adaptive immunity mechanisms

Thus far, it was clear that the gonococcus could evade host innate immunity through a variety of mechanisms. Gonococci also evaded host adaptive immunity through direct effects on antibody producing cells: for example, gonococci were shown to kill CEACAM1-expressing human B cells in vitro, which inhibited antibody production [370]. But the most important mechanisms of evasion centered on the ability of the gonococcus to suppress adaptive immune responses through effects on antigen presenting cells (APCs) such as macrophages and DCs.

Gonococcal interactions with macrophages may have contributed significantly to the suppression of adaptive immunity. Following phagocytosis, gonococci could induce a tolerogenic or M2-like macrophage phenotype [281] characterized by increased production of IL-10 and TGF-β and enhanced expression of the immunosuppressive ligand PD-L1. These changes impaired antigen presentation through reduced expression of MHC class II and co-stimulatory molecules and diminished the capacity of macrophages to stimulate CD4+ T-cell proliferation. Consequently, macrophage responses favored regulatory pathways rather than the development of protective Th1- and Th2-mediated immunity. Together with gonococcal induction of IL-10- and TGF-β-dependent regulatory networks, these effects were likely to contribute to the weak immunological memory and susceptibility to reinfection that were characteristic features of gonorrhea [281,371,372].

In gonorrhea, DCs were also important because they interacted with gonococci, produced cytokines, and influenced T-cell responses. DCs were specialized white blood cells that acted as sentinels of the immune system that presented antigen to T lymphocytes and thus formed a crucial bridge between the innate and adaptive immune system [373]. An early study showed that phagocytosis of gonococci by human DCs in vitro was mediated by the Dendritic Cell-Specific Intercellular Adhesion Molecule-3-Grabbing Non-Integrin (DC-SIGN) receptor and did not involve Opa-CEACAM1 interactions [374] (Figure 1). However, only a LOS *lgtB* mutant of the gonococcus was recognized by DC-SIGN and subsequently phagocytosed, which suggested that LOS variation may have evolved as a mechanism for gonococci to avoid phagocytosis by DCs. Thus, avoidance of DC phagocytosis was likely to reduce the induction of effective adaptive immune responses and immunological memory.

Another study showed that gonococcal LOS variation allowed for the selective manipulation of human DC function, thereby shifting subsequent immune responses in favor of bacterial survival [335]. In this study, different stable gonococcal LOS phenotype variants targeted a different set of receptors on human DCs, including the C-type lectins Macrophage Galactose-type Lectin (MGL) and DC-SIGN (Figure 1). Notably, both MGL and DC-SIGN were locally expressed at the male and female genital area [335]. Targeting of these different C-type lectins with the LOS variants altered the DC cytokine secretion profiles and induced distinct adaptive CD4(+) T helper responses. Whereas *N. gonorrhoeae* variant A with a terminal N-acetylglucosamine on its LOS was recognized by DC-SIGN and induced significantly more IL-10 production, the LOS phenotype C, carrying a terminal N-acetylgalactosamine, primarily interacted with MGL and skewed immunity toward the Th2 lineage. These observations suggested that LOS variation allowed for the selective manipulation of DC and shifted immune responses in favor of bacterial survival.

Using primary, murine bone marrow-derived DCs and lymphocytes, Zhu et al. [365] showed that DCs exposed to gonococci did not elicit antigen-induced CD4+ T lymphocyte proliferation. Exposure to pathogen upregulated several secreted and DC cell surface proteins with immunosuppressive properties, i.e. IL-10 and PD-L1. Gonococci inhibited DC-induced proliferation of human T-cells, and human DCs upregulated similar immunosuppressive molecules. Thus, gonococci could suppress the development of adaptive immune responses through interactions with host APCs. Subsequently, PorB present in shed OMVs was identified as the factor involved in suppressing the capacity of APCs to induce CD4+ T cell proliferation [375]. In addition, *N. gonorrhoeae* evaded the development of effective adaptive immunity by using Opa proteins to bind CEACAM1 on human DCs, which impaired their maturation and reduced their ability to activate T cells. As a result, infected DCs were less able to stimulate antigen-specific immune responses, including HIV-1-specific memory cytotoxic T lymphocytes. This helped to explain, in part, why gonorrhea often failed to generate protective immune memory and exacerbated HIV infection [376].

In another study, the direct interactions of gonococci with human DCs in vitro induced DC maturation and stimulated the production of prostaglandin E2 (PGE2), IL-23, and other pro-inflammatory mediators [377]. This PGE2–IL-23 axis was shown to drive robust Th17/IL-17 responses from memory CD4^+^ T cells, leading to marked local inflammation. However, despite appearing phenotypically activated, gonococcus-exposed DCs were functionally altered and promoted a Th17-biased response at the expense of protective adaptive immunity. Importantly, together with previous observations that Opa-expressing gonococci impaired DC maturation and antigen-presenting function through CEACAM1 engagement, these findings suggested that gonococci manipulated DCs to favor inflammatory yet non-protective immune responses. Rather than inducing classical immunosuppression, the bacterium caused immune deviation, skewing immunity toward Th17-driven inflammation while suppressing efficient Th1 responses, suppressing cytotoxic T-cell activation, and suppressing the development of long-lasting immunological memory. This mechanism may have contributed to persistent gonococcal infection, repeated gonococcal reinfection, and impaired immunity to co-infecting pathogens such as HIV.

## How is the gonococcus naturally cleared in lower genital tract infections?

What was abundantly clear by now was that the gonococcus was exceptionally adept at avoiding and suppressing host innate immunity and actively blunting and misdirecting adaptive immunity. Yet, uncomplicated gonococcal infection in humans may resolve spontaneously, and a summary of the key mechanisms and features, explained in the preceding text, is provided in Table 1. Briefly, the cumulative evidence suggested that clearance was driven primarily by innate immune mechanisms. The hallmark of gonococcal infection was the intense recruitment of neutrophils, which gave rise to the characteristic mucopurulent discharge. Neutrophils phagocytosed gonococci and exposed them to oxidative and non-oxidative killing mechanisms, while simultaneously releasing antimicrobial mediators such as lysozyme, defensins, lactoferrin, proteases, and calprotectin. Although the gonococcus has evolved numerous strategies to survive within this hostile environment, the sustained influx of neutrophils and the associated inflammatory damage eventually favored bacterial elimination. This role of neutrophil-dominated inflammation was a central feature of host defense against gonococcal infection.

**Table 1.** Mechanisms driving resolution of uncomplicated gonococcal infection.

Host response	Key features	Outcome
1. Neutrophil driven inflammation (central mechanism)	Purulent discharge composed largely of neutrophils Neutrophils phagocytosed gonococci and exposed bacteria to oxidative and non- oxidative killing. Neutrophils released c-type lysozyme, defensins, lactoferrin, proteases, and calprotectin	Sustained neutrophil flux and epithelial shedding eventually tipped the balance towards bacterial elimination in many uncomplicated cases.
2. Antimicrobial factors in mucosal fluids	Gonococci were continuously exposed to innate antimicrobials at mucosal surfaces, e.g. c-type lysozyme damaged cell walls, induced cationic stress Defensins (α and β) disrupted membranes Lactoferrin and transferrin sequestered iron AMPs, Secretory Leukocyte Protease Inhibitor (SLPI), cathelicidins, complement proteins	These did not rapidly sterilize infection, but increased envelope stress, synergized with neutrophils and selected for resistance mechanisms that cost fitness.
3. Epithelial exfoliation and turnover	Especially in the cervix, urethra and rectum. Inflammation caused desquamation of infected epithelial cells and the loss of gonococcal adhesion niches (pili, Opa/CEACAM, etc.)	As *N. gonorrhoeae* acted as a mucosal pathogen and was generally non-invasive and thus surface attached, epithelial turnover was an underappreciated but important clearance mechanism.
4. Nutritional immunity (iron and metal restriction)	During inflammation, Host upregulated lactoferrin, calprotectin, hepcidin Iron, zinc, and manganese availability fell	Although the gonococcus had transferrin binding proteins and lactoferrin receptors, these systems were not infinitely efficient, and growth was constrained, especially under concurrent immune pressure.
5. Limited and ineffective adaptive immunity	Clearance was not driven by protective adaptive immunity. Adaptive immunity failed because of Antigenic and phase variation Active suppression of Th1/Th2 responses and of B cell memory; skewed towards a Th17 dominant inflammation	Consequences were that Antibodies were produced but poorly protective Little or no immune memory Reinfection was common, even with the same strain
6. Asymptomatic infections and spontaneous clearance	In many women (and some men), infection was asymptomatic, with the likely contributors being Lower bacterial burden Baseline mucosal immunity Menstrual cycle–associated epithelial turnover Microbiome effects (especially vaginal *Lactobacilli*)	Clearance may occur over weeks–months

In parallel, gonococci were continuously exposed to a range of antimicrobial factors present within mucosal secretions. Lysozyme, defensins, secretory leukocyte protease inhibitor (SLPI), cathelicidins, complement components, and iron-sequestering proteins such as lactoferrin and transferrin, collectively imposed substantial envelope and nutritional stress on the organism. While these innate defenses rarely sterilized infection rapidly, they acted synergistically with neutrophils to constrain bacterial growth and may have imposed fitness costs on mechanisms required for resistance and persistence.

Another important, but often underappreciated, mechanism of clearance was epithelial turnover. As a predominantly surface-associated mucosal pathogen, *N. gonorrhoeae* relied on interactions between pili, Opa proteins, and their host epithelial receptors to maintain colonization. Inflammation-induced epithelial exfoliation and shedding removed infected cells and disrupted these adhesion niches. Consequently, continuous epithelial renewal within the urethra, cervix, and rectum may have physically dislodged attached bacteria and progressively reduced the available habitat for colonization.

Host-imposed NI further contributed to bacterial clearance. During inflammation, proteins such as lactoferrin, calprotectin, and hepcidin restricted the availability of essential metals, including iron, zinc, and manganese. Although the gonococcus possessed sophisticated systems for acquiring these nutrients directly from host proteins, these mechanisms were not completely efficient. As inflammation intensified and nutrient availability declined, bacterial replication became increasingly constrained, particularly when combined with neutrophil-mediated and AMP-mediated stress.

Taken together, spontaneous resolution of uncomplicated gonorrhea was best viewed as the cumulative outcome of prolonged innate immune pressure rather than the establishment of effective adaptive immunity. Sustained neutrophilic inflammation, antimicrobial factors in mucosal secretions, epithelial exfoliation, and NI progressively eroded bacterial fitness and colonization capacity until the balance shifted in favor of the host. This process might occur over weeks to months, particularly in asymptomatic infections where lower bacterial burden, baseline mucosal defenses, epithelial turnover associated with the menstrual cycle, and potentially protective microbiome effects, such as those mediated by vaginal lactobacilli, further promoted clearance.

Indeed, to take this further, one could consider that gonococci shared similarities with commensal *Neisseria* species that rather modulated host immune homeostasis to promote prolonged colonization, instead of being rapidly cleared by the immune system [378]. *N. gonorrhoeae* employed extensive antigenic and phase variation and actively manipulated host immune responses, suppressing effective Th1- and Th2-mediated immunity and impairing the development of durable B-cell memory. Thus, gonococcal infection did not induce specific protective immunity or immune memory in mice [379]. In addition, recurrent infections could occur in men as soon as 21 days after an initial infection [336]. Only invasive infections such as pelvic inflammatory disease (PID) induced partial natural protective immunity [380]. Indeed, nasopharyngeal colonization of humans with commensal *Neisseria* species, e.g. *N. lactamica* did not stimulate mucosal T or B cell memory responses [381], comparable to the outcome of uncomplicated gonococcal infection. Thus, could one speculate that the obligate gonococcus restricted to the lower reproductive tract (LRT) is evolving into a commensal organism?

## Dissemination of the gonococcus from the genital tract

Gonorrhea was typically uncomplicated and restricted to the lower genital tract. However, in approximately 10–25% of untreated women, gonococci could ascend into the upper reproductive tract (URT) and the host response to this ascending infection was manifested as the clinical syndrome of pelvic inflammatory disease (PID). PID left patients with long-term and/or permanent sequelae such as chronic pelvic pain, Fallopian tube (FT) damage, tubo-ovarian abscesses, endometritis, and increased the risk of ectopic pregnancy and infertility [382]. Charting the ascension into the URT of women from the ecto/endocervical site of infection, gonococci must engage sequentially with the mucosae of the endometrium and FT, prior to contact with the ovaries and access to the peritoneum. In addition, gonococcal infection could be complicated by the ability of the organism to disseminate away from the lower genital tract and seed other anatomical sites, generally via the blood (Figure 6).

**Figure 6.**
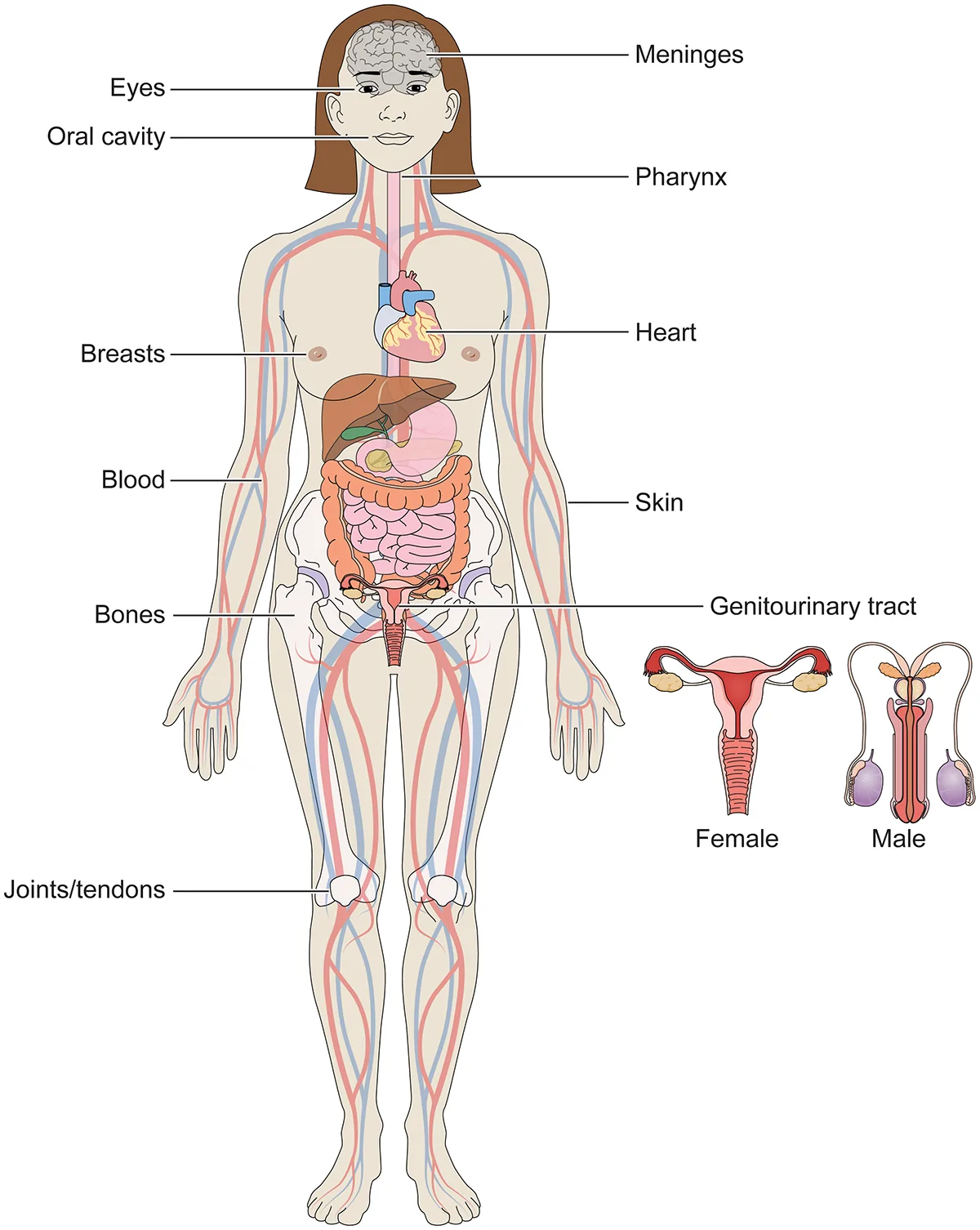
A cartoon showing the sites of the human body that can be colonized by *N. gonorrhoeae*. Most of these extra-genital sites were accessed by the gonococcus via the blood; infection of the eyes and skin could occur by direct transmission to these surfaces.

A mouse model has been developed to mimic URT infection, and it demonstrated that the reproductive cycle was a pathogenic determinant during murine PID, and by inference, human PID [383]. In this model, direct transcervical inoculation of gonococci into the uterus of mice was used to establish URT infection. Notably, mice infected during the diestrus stage showed extensive gonococcal penetration into the submucosa, severe inflammation, and clinical signs reflecting discomfort. Conversely, infection during the estrus stage showed only modest effects, and anti-gonococcal antibodies were produced only during diestrus. In addition, a chemokine-dominant response was observed during diestrus, with greater activity of granulocyte adhesion and diapedesis regulators, a specific induction of type-1 interferon signaling pathways and greater pathology [384]. Induction of the latter pathways was suggested to be host detrimental.

Like the URT model of infection, it was worth noting that the mouse model of intravaginal (LRT) gonococcal infection, which has been used more extensively for studying pathogenesis and for vaccine and drug development, was an imperfect mimic of human infection. The main reasons for this were because the gonococcus was a strict obligate human pathogen and infection of mice with piliated and Opa-expressing gonococci was unnatural or inefficient, due to the lack of specific receptors on the mouse epithelium (unless transgenic animals were used). To achieve infection required treating mice with estradiol to make the epithelium more permissive, the use of antibiotics to reduce normal flora and the use of high inocula of bacteria [385]. Even then, infection was short-lived and mainly reflected surface colonization of the LRT, and ascending or disseminating infection could not be developed. Thus, although the mouse was useful for studying general principles, e.g. of inflammation and some aspects of colonization, it was compromised because it failed to capture true human-specific receptor interactions, true tissue tropism and natural immune evasion strategies.

### Gonococcal interactions with the endometrium

Hypothetically, gonococci could ascend to the endometrium via 1) direct vertical physical transmission during sex, including on sperm-bound gonococci, 2) by bacteria sloughing from macrocolonies or biofilms established in the cervix, or 3) perhaps by escaping from inside neutrophils in the pus exudate that was not contained in the lower genital tract but infiltrated into the endometrium. In addition, gonococcal ascent could be aided during menstruation when cervical mucus was less viscous, and the mucus plug was lost. Histopathologically, endometritis in women was characterized by the presence of neutrophils in the endometrial surface epithelium and within gland lumens, a dense subepithelial stromal lymphocytic infiltration, the presence of stromal plasma cells, and germinal centers that contained transformed lymphocytes [386]. Immunohistochemical characterization of the endometrial leukocyte subpopulations from 37 control women and 45 women infected with *C. trachomatis, N. gonorrhoeae*, or *T. vaginalis* showed that endocervical infection was associated with significant increases in endometrial T cells, B cells, plasma cells, and Polymorphonuclear Leukocytes (PMNLs) [387]. In a mouse model of infection, evidence of endometritis was demonstrated by detection of gonococci on the mucosal surface and by a PMNL infiltration that contained intracellular gonococci, in both the epithelium and the lamina propria [388]. There were scattered microabscesses in subepithelial tissues and penetration of the underlying muscular layer.

In laboratory studies, gonococci adhered to and invaded Hec1B cells in vitro, which resulted in cell death [115]. Invariably, pili and Opa were involved in this process with Hec-1B [389] and primary human endometrial [390] cells. Studies with Hec-1B cells suggested that transferrin also increased gonococcal adherence [391]. In addition, deletion of the proximal glucose residue of the α-OS chain of LOS significantly impaired the invasion of Chang, HEC-1B and ME-180 cells in vitro [392], demonstrating the importance of this residue for gonococcal invasion of host mucosae. Surface expression of L12, a gonococcal “hCG (human chorionic gonadotropin)-like” protein, was shown also to mediate invasion and transcytosis of Hec1B cells via the lutropin receptor [393].

Using human endometrium organ culture and human primary endometrial epithelial cell models, gonococci appeared to use multiple mechanisms to associate with the endometrium [394,395]. Gonococci attached to cilia but were not observed associated with the plasma membrane of ciliated epithelial cells or internalized into ciliated epithelial cells. The organism could be found in intracellular vacuoles in secretory epithelial cells. Intimate interactions included colocalization with the ASGP-R, CEACAM and CR3, and the formation of lamellipodia and ruffles and microvillus engagement [394]. Several studies in vitro with primary human endometrial cells and explant models also reported that gonococcal infection induced a cytokine/chemokine-rich inflammation [390,395]. Furthermore, mucosa-associated invariant T (MAIT) cells present in the endometrium from pre- and postmenopausal women, and in decidua from first-trimester pregnancies, responded to gonococci in functional experiments in vitro [396]. These observations provided additional evidence of mucosal defense. Alongside inflammation, gonococci suppressed the viability of human endometrial epithelial cells in vitro and induced apoptosis: a role for TLR2 and TLR4 recognition was described for this process, which demonstrated, again, the innate recognition of peptidoglycan fragments and LOS, respectively [397].

### Gonococcal interactions with the Fallopian tubes

Hypothetically, ascension to the Fallopian tubes (FTs) could again be through vertical physical transmission during sex, with or without binding to sperm, via uterine peristalsis, and an inflammation-induced barrier breakdown that enabled gonococci to access deeper tissues. The FT was especially vulnerable, as the tubal epithelium contained a large proportion of ciliated cells that were targeted by gonococci, and the mucosal barrier was relatively delicate. The pathogenesis of salpingitis, defined as the inflammatory response in the FT caused by gonococcal infection, was reviewed extensively by Lenz and Dillard [398] and did not need detailed repetition here. It was useful however, to highlight the similarities between colonization of the FT mucosa and other mucosae, i.e. mediated by gonococcal pili and Opa, and the roles of LOS and peptidoglycan to induce inflammation. Early work describing laparoscopic sampling of gonococci from human FTs recovered a higher proportion of Opa- over Opa+ gonococci. In another contemporaneous salpingitis study, transparent colonies (>95%) were mostly recovered, while ≥ 50% of lower genital tract isolate colonies were opaque [399]. In addition, Pil+ Opa- (transparent) gonococci bound 10–100-fold stronger to FT explants in vitro than Pil+Opa+ (opaque) bacteria [400].

The mechanics of gonococcal interactions with the FT owed a great deal to the pioneering studies from McGee and colleagues [401–409] and were as follows: 1) piliated and non-piliated gonococci bound preferentially to secretory (non-ciliated) cells and invaded and exited quickly without residing within these cells; gonococcal infection also disrupted inter-epithelial cell junctions to facilitate invasion [410]; 2) primary damage occurred to ciliated epithelial cells with cell sloughing, which was attributed to LOS and peptidoglycan fragments [405–407]; 3) gonococci were recognized by host PRRs, including TLRs and cytosolic NOD1 and NOD2, RIG-1 (retinoic acid-inducible gene 1) and MDA5 (melanoma differentiation-associated protein 5) [398], with attendant activation of intracellular signaling pathways; 4) TNF-α was produced as the cardinal cytokine causing ciliary cell death; in females, LOS [411] and peptidoglycan fragments contributed to salpingitis [405,412] and TNF-α–induced apoptosis [413]; 5) several apoptosis-related genes were expressed by gonococcal-infected primary FT epithelial cells in vitro [414]; 5) other cytokines/chemokines produced by the FT included IL-1β, IL-6, IL-8, MCP-1, MIP-1β and GM-CSF [415]; 6) production of IL-17C by epithelial cells induced a damaging inflammation in the FT [416]; 7) matrix metalloproteinases (MMP2 which accumulated intra-epithelial cells, MMP9 that was secreted, and MMP8 that increased in gonococcal-infected FT explants) were induced, and these damaged epithelial cells [417,418]; 8) gonococci interacted with immune cells resident in the FT, which included DCs, macrophages and T-cells, all of which were manipulated to suppress the development of an adaptive immune response; 9) gonococci interacted with neutrophils that migrated into the infected FT [419], and these cells produced LTB4; 10) gonococci also interacted with DCs, which produced PGE2 [377]. High levels of both LTB4 and PGE2 were recovered in the peritoneal fluid of women with acute PID salpingitis [420].

Although TNF-α-mediated apoptosis of FT epithelial cells was recognized as a primary host defense mechanism to prevent gonococcal colonization, one study showed that FT epithelial cell-associated gonococci had evolved a mechanism to protect the cells from undergoing this apoptosis. This modulation of the host innate response may have contributed to establishing infection [421]. Evidence of further modulation was the fact that pili inhibited several key elements of the initial inflammatory response, which facilitated sustained infection of FT explants in vitro [422]. This sequence of events seemed well-established, but other aspects of FT infection were equivocal; for example, a role for NO in epithelium damage was unclear [423] and required further investigation.

Thus, the biological significance of gonococcal infection of the female URT (endometrium and FT) was that it represented the transition from localized mucosal colonization to invasive inflammatory disease. In both compartments, the cumulative outcome was a balance between host attempts to clear infection and bacterial mechanisms that promoted survival, with excessive inflammation producing the observed clinical outcomes. If untreated, these responses collectively resulted in pain, inflammation in the endometrium and FT, blockage of the FT, epithelial exfoliation, pelvic adhesion and, eventually, collateral damage to the human female genital tract [424].

### Gonococcal interactions with blood

On the rare occasions that gonococci entered the bloodstream during DGI, their survival depended primarily on avoiding complement-mediated killing. Indeed, DGI-associated isolates were generally more serum-resistant than strains causing localized mucosal infection [425]. Gonococci further resisted clearance by neutrophils through modulation of CEACAM-dependent interactions, suppression of oxidative responses, and resistance to intracellular killing. Concurrent antigenic and phase variation of surface structures, together with suppression of APC function and adaptive immune responses, allowed the gonococcus to evade both innate and acquired immunity. This facilitated hematogenous dissemination and the development of DGI. All these mechanisms are described in the appropriate sections above.

### Disseminated gonococcal infection (DGI) of other anatomical sites

Although gonococci had mechanisms to evade recognition and attack from innate immune cells and complement, they generally had a reduced capacity to survive systemically and rarely left their normal colonization niches [426]. However, gonococci could cause primary infections of other anatomical sites due to direct interaction of the pathogen [427] (Figure 6). Indeed, possible receptors for piliated gonococci were expressed on diverse human tissues such as the cervix, endometrium, cornea, intestine, stomach, mid-brain, and meninges [428]. Two forms of DGI were distinguished: the bacteremic “arthritis–dermatitis” syndrome and the localized suppurative (septic) arthritis. Conceptually, the bacteremic form was an early disseminated, immune/inflammatory phase with bacteremia and metastatic features but little frank pus in joints. The suppurative form represented a later/localized stage, where bacteria had seeded the joint(s) and produced classic septic arthritis.

### Bacteremic “arthritis–dermatitis” syndrome

As noted already, gonococcal strains associated with DGI were more serum resistant than strains isolated from localized infections [425]. The presence of a variable genetic island in gonococci was associated with the ability of DGI-associated gonococcal isolates to become systemic [12]. This island bestowed gonococci with a serum resistance locus and encoded also for a peptidoglycan hydrolase. Expression of the latter may have correlated with increased peptidoglycan-cytotoxin production and thereby enhanced pathogenicity by increasing systemic survival of the gonococcus [429]. In addition, gonococcal porin may have played a possible role in disseminating infection. It has been reported that gonococci expressing PorB.IA were frequently isolated from patients with DGI, mimicking systemic bloodstream infections by triggering efficient adherence and invasion of epithelial and other cell types under low phosphate conditions. This was shown using human cell lines in vitro, including Chang conjunctival epithelial cells, HUVEC and HeLa cells, wherein the human heat shock glycoprotein Gp96 was identified as a surface receptor for PorB.1A [430]. Depletion of Gp96 from cells prevented adherence but triggered gonococcal invasion, which was dependent on interaction with the scavenger receptor SREC (Scavenger Receptor expressed in Endothelial Cells). Thus, Gp96 appeared to act as an anti-invasion molecule and SREC as a receptor enabling invasion of host cells by highly invasive disseminating gonococci [430].

Fatality was a potential outcome from DGI, and the typical sequelae associated with bacteremic DGI were infectious arthritis-dermatitis (rash), and these resulted mainly from blood dissemination of gonococci from primary sexually acquired mucosal infection [431]. The presentations of tenosynovitis, migratory polyarthralgia (non-purulent) and cutaneous lesions were commonly associated with gonococcal bacteremia, but may have been an indicator of DGI too [432]. Cutaneous lesions included abscesses of the scalp [433], cellulitis, petechiae, purpuric macules, necrotizing fasciitis, and vasculitis [434]. Scalp abscesses with necrosis could become a focus for DGI [435] and direct inoculation of disrupted skin with gonococci has been reported, rarely, to induce mastitis in the breast [427]. Other rare manifestations of DGI included endocarditis [436,437], myocarditis [438,439], pericarditis [440], osteomyelitis (via extension from an infected joint) [441], pyomyositis [442], acute hepatitis [443], classical Fitz-Hugh–Curtis syndrome perihepatitis [444], meningitis [445,446], Waterhouse–Friderichsen syndrome [447], and adult respiratory syndrome [448,449]. Although true fulminant sepsis was extremely rare in gonococcal DGI [450–452] compared with the meningococcus, the same fundamental steps – escape from local control, serum resistance, and immune evasion – caused systemic disease.

### Suppurative (septic) arthritis

In suppurative (septic) arthritis, the key distinguishing features were localized infection of one (or few) joints, which were typically monoarticular or oligoarticular, and the production of a purulent synovial fluid (a true septic arthritis) [453]. The knee, wrist, ankle, and elbow were commonly affected, and there were minimal or absent skin lesions and tenosynovitis, compared with the bacteremic form. Once again, the pathogenesis of gonococcal arthritis was multifactorial. From the bloodstream, gonococci seeded the vascular synovial membrane, with bacterial colonization leading to a host inflammatory response and the development of a septic joint. Bacterial entry was facile as the synovial membrane had no limiting basement plate under the well-vascularized synovium [454]. Moreover, the synovial joint was a closed space and hence there were low fluid shear conditions, and these conditions enabled bacterial adherence. The gonococcal factors that mediated interactions with the host were likely many of those described above. The host response was inflammatory, with the production of cytokines (IL-1β, IL-6) by synovial cells and the influx of neutrophils and macrophages, which together with synoviocytes, released additional cytokines (e.g. TNF-α, IL-8, GM-CSF, IL-1β and IL-6) [454]. Joint destruction was common, if untreated.

### Gonococcal invasion of the subarachnoid space (SAS)

Additional to synovial joints, the other compartment that could be invaded by the gonococcus passing directly from the blood was the cerebrospinal fluid (CSF)-filled subarachnoid space (SAS) of the meninges. In the case of the SAS, the mechanism of entry of the gonococcus was unknown but hypothetically was likely to be like that used by the meningococcus, i.e. requiring bacteremia [455]. The portal of entry could again be the thin-walled vessels within the SAS, and the host response was a characteristic cytokine/chemokine-rich inflammation with neutrophil influx. Like the meningococcus, virulence factors involved in adhesion to vessel endothelium and to the leptomeninges included the pilus and Opa protein [456], and cytokine production was again likely induced by LOS [455]. A role for the gonococcal MafA protein in invasion of the SAS was postulated on two strands of evidence: 1) the meningococcal variant MafA1 (NMB0375) was upregulated in the presence of glucose, and the *hexR* gene that was part of the HexR regulon accounted for some of this glucose-responsive regulation, and *hexR*-deficient meningococci did not establish successful bacteremia in an infant rat model of infection [457]; 2) meningococcal MafA adhered to human brain microvascular endothelial cells in vitro [458]. Both bacteremia and adherence to vessel endothelia were pre-requisites for translocation of bacteria across the human blood-CSF barrier to cause meningitis [455]. Similar functions of the gonococcal MafA protein could be suggested, given the high degree of amino acid sequence similarity between the gonococcal and meningococcal proteins.

### Gonococcal infection of the oral cavity and nasopharynx

Sexual transmission of gonococci from the urogenital tract commonly produced oral and nasopharyngeal infections [459], including pharyngitis [460], tonsillitis [461], stomatitis (inflammation of the oral mucosa) [462], infection of the parotid gland [463] and ulceration of the tongue [464]. The key mechanistic steps in oral/nasopharyngeal gonococcal infection were common to infection of the reproductive tract mucosae, sequentially involving mucosal inoculation, i.e. transmission by oral contact, adhesion, colonization and microcolony formation on mucosal surfaces, cell entry, and host immune engagement with an inflammatory response and neutrophil influx. These infections remained localized, although DGI from a primary pharyngeal infection has been described in a prepubertal child [465]. Pharyngeal infection was of particular concern for the emergence of AMR in gonococci, and pharyngeal gonorrhea enabled the spread of resistant gonococci and potentially led to untreatable infections [466].

### Gonococcal infection of the eye

The eye was probably the only place where the gonococcus could be aggressively invasive on a mucosal surface. The outcome was defined as gonococcal conjunctivitis/ophthalmia and ophthalmia neonatorum when it occurred in neonates. The mechanisms were quite well solved. Transmission routes in adults included autoinoculation from genital secretions (hand to eye) [467,468] and direct inoculation of neonates at birth by vertical transmission of infectious vaginal secretions, or more rarely with delivery via cesarean section [469,470]. Once gonococci reached the conjunctiva, events would unfold rapidly, principally because the structure of the conjunctival epithelium was non-keratinized, highly vascular and receptor-rich [471], and therefore permissive to infection [472]. Gonococcal adhesion to conjunctival epithelial cells used the same mechanisms and virulence factors as with other epithelia (i.e. with pili, Opa), but they efficiently invaded these cells and involved the cornea [473]. The course of the disease and its pathology have been studied extensively in experimental infection of the rabbit eye [474].

The more precise mechanisms of gonococcal interactions with conjunctival cells at the cellular level have been studied extensively in vitro using Chang conjunctival epithelial cell cultures, and these studies unraveled the involvement of Opa. Opa protein established binding to HSPG side chains (HSGAGs) of surface-associated HSPGs expressed on epithelial cells, including Chang cells [76,77,475]. HSPG-dependent gonococcal invasion of Chang cells depended on the activation of phosphatidylcholine-dependent phospholipase C (PC-PLC) and acidic sphingomyelinase (ASM), which resulted in the generation of the second messengers, diacylglycerol (DAG) and ceramide, respectively [476]. Recent studies in vitro with a different cell line, in this case a telomerase-immortalized human corneal epithelial cell line (hTCEpi), showed that gonococcal adherence depended on the functional pilus in the early stages of infection. The bacteria formed bacterial microcolonies and biofilms on the cell surface that were tissue destructive [477].

Corneal involvement was rapid, and again, LOS-driven inflammation resulted in neutrophil recruitment and a copious purulent discharge [472]. The consequence of gonococcal-induced IL-8 secretion in vivo was neutrophil chemotaxis, and induced TNFα would be involved in corneal tissue destruction. On the ocular surface, gonococci would encounter a tear film rich in secretory IgA and lysozyme. The gonococcus would be well adapted to this environment through production of the IgA1 protease that cleaved mucosal antibodies. In addition, expression of the lysozyme inhibitors ACP and SliC protected against tear-derived antimicrobial activity, and LOS sialylation suppressed neutrophil activation and enhanced resistance to innate immune killing [172,209,260,333]. These mechanisms likely contributed to the ability of gonococci to persist on the ocular surface and to exploit the intense inflammatory response characteristic of gonococcal keratoconjunctivitis.

Gonococcal infection was rapidly destructive, particularly for newborn eyes, and led to corneal scarring and blindness. Corneal ulceration could occur with probable perforation of the globe and a panophthalmitis [435] and the infected conjunctivae occasionally served as a portal of entry for gonococci to induce the panoply of DGI manifestations, including septicemia, meningitis, and arthritis [478]. In adults, unlike more common forms of bacterial conjunctivitis, gonococcal infection caused corneal perforation and permanent blindness within hours, if not treated or repaired surgically [479].

### Dissemination in the male

Above, we focused on dissemination of infection in the female, but it did occur also in males. Gonococci moving away from the urethra engaged with several local organs and induced inflammation. Gonococci induced 1) epididymitis in the epididymis, characterized by scrotal pain and swelling, which affected fertility [480,481]; 2) prostatitis in the prostate, with symptoms of pelvic/perineal pain, dysuria, and painful ejaculation [482]; 3) orchitis in the testicles, which was rare but could occur via epididymitis [483,484]; and 4) peri-urethral abscesses, which were localized collections of pus that formed near the urethra [485].

Male infertility was a recognized complication of untreated gonococcal infection, chronic infection, or episodes of recurrent infections. Ascending infection with epididymitis, epididymo-orchitis, prostatitis, and urethral fibrosis, ultimately impaired spermatogenesis and sperm transport through inflammatory damage and scarring of the reproductive tract [486–488]. Chronic gonococcal infection was associated with reduced semen quality, oxidative stress, and the development of obstructive azoospermia [488,489]. The pathogenesis of dissemination through the bloodstream, seeding of other sites, the interactions of virulence factors with host cells, and the development of inflammation followed the general principles described above.

## Treatment and prevention of gonococcal infections

Gonococci have developed resistance to every single class of antibiotic used to treat infection. Developing resistance was a virulence trait that favored bacterial survival and in the gonococcus was both plasmid- and chromosomally-mediated. The mechanisms were well-characterized and needed no further details here. The reader was directed to several recent reviews on gonococcal AMR for more information [490–494].

Now afforded the moniker of “superbug,” vaccination was increasingly seen as an optimal strategy to prevent gonorrhea, and it has been considered in many excellent reviews since the Summary and Recommendations from the National Institute of Allergy and Infectious Diseases Workshop in 2016 [490,495–499]. Central to vaccine development was an understanding of the pathogenesis of gonorrhea and the nature of the virulence factors and potential antigens that could be used to generate immunity [500]. The list of potential antigens was ever-increasing, but gonococcal vaccine development faced the considerable bottleneck in not having a correlate of protection, and whether antibody and/or T-cell mediated immunity at the mucosae was the goal. Moreover, even if experimental vaccines met biological criteria such as the generation of bactericidal/opsonophagocytic activity and active protection and passive antibody-mediated protection in the mouse model of gonorrhea, translation to the human has largely failed [501].

### The effect of meningococcal Bexsero/4CMenB on gonorrhoea

A dependence on the vaccine Bexsero/4CMenB, which contained a meningococcal OMV (MenZB) and three recombinant meningococcal proteins (fHbp, factor H binding protein; NadA, Neisserial adhesin A; NHBA, Neisserial Heparin Binding Antigen), would be misplaced for the generally sexually active heterosexual community. The rationale for its use came anecdotally from Cuban and Canadian experiences and from the retrospective case-control study in New Zealand of vaccines containing meningococcal OMV [502,503]. In the New Zealand study, the estimated effectiveness of the MeNZB OMV vaccine against gonorrhea after adjustment for ethnicity, deprivation, geographical area, and sex was 31% (95% CI 21–39) [503]. Furthermore, after similar adjustments, the effectiveness of MeNZB against hospitalization caused by gonorrhea was estimated to be 24% (95% CI 1–42%) [504]. Regardless, on 10 November 2023, the UK’s Joint Committee on Vaccination and Immunization recommended starting a targeted Bexsero vaccination program against gonorrhea for individuals at higher risk, notably men-having-sex with men, trans women/gender-diverse individuals assigned male at birth, heterosexuals of similar risk, and sex workers with condomless sex (JCVI advice on the use of meningococcal B vaccination for the prevention of gonorrhea – GOV.UK). Implementation began on 1 August 2025, alongside mpox, hepatitis A/B, and HPV vaccines, where appropriate. Modeling has suggested that efficacy of the vaccine could be anywhere between 20% and 47% [505–510], although there were significant caveats with its use. It will be interesting to see how adaptable the gonococcus becomes to immune pressure from this vaccine. A recent systematic review and meta-analysis of 12 studies eligible for qualitative analysis and 6 studies eligible for quantitative analysis (of 4CMenB alone), of the effectiveness of meningococcal OMV vaccines against gonorrhea, reported that 1) vaccine effectiveness (VE) derived from the pooled analysis was 38% (95 % CI:22–50%; I^2^ = 55 %), 2) VE was 41% (OR = 0.59; 95 % CI:0.46–0.76; I^2^ = 44 %) for 4CMenB vaccine alone, 3) complete vaccination might offer 24% greater protection compared to partial vaccination (OR = 0.76; 95 % CI:0.31–1.85; I^2^ = 86 %), and notably 4) VE declined in 2–3 years after vaccination [511].

### Identifying cross-species protective OM antigens

Thus, the epidemiological evidence showed that meningococcal OMV vaccines provided only moderate protection against gonorrhea, and that cross-protection was likely multifactorial and mediated by different humoral and cellular immune responses [512]. However, this moderate protection might only be observed among heterosexuals: a recent report of the results from an open-label, randomized controlled trial carried out between July 2021 through May 2023, to evaluate the efficacy, safety, and immunogenicity of meningococcal 4CMenB vaccine against gonococcal infection in gay and bisexual men, found no evidence of protection against gonococcal infection [513]. The efficacy data were recently published by Seib et al. [514]. Gonorrhea incidence rates were almost identical between the vaccine and placebo groups, at 48.1 and 47.8 cases per 100 person-years, respectively, corresponding to 160 infections among 296 vaccinated participants and 155 infections among 291 placebo recipients. This resulted in an incidence rate ratio of 1.01 (95% CI, 0.80–1.26; *p* = 0.97), indicating an estimated VE of −0.5% (95% CI, −26.2% to 19.9%). For symptomatic infection, VE was 5.5% (95% CI, −56.0 to 42.8). For asymptomatic infection, VE was −6.4% (95% CI, −47.5 to 23.2), and −20.0% (95% CI, −90.2 to 23.9), −1.2% (95% CI, −33.0 to 23.0), and 2.6% (95% CI, −27.7 to 25.7) for urogenital, anorectal, and oropharyngeal infection, respectively. There were reports of serious adverse events in 4.7% of the participants in the 4CMenB group and in 2.8% of those in the placebo group. It should be noted that this was a limited study, impacted by the COVID-19 pandemic, and more authoritative data on the efficacy of this vaccine in high-risk groups should come from the UK program.

Issues with 4CMenB vaccine were also evident in the mouse gonorrhea model, where vaccination did not induce consistent protection, and gonococci were cleared from only a subset of immunized mice [515]. This partial protection was observed even though 4CMenB induced 1) high levels of anti-gonococcal antibodies in serum and the vaginal lumen, 2) a broad cytokine response that covered Th1 (IL-2, TNFα, IL-12p40), Th2 (IL-4, IL-5, IL-13), T_reg_ (IL-10), and Th17 (IL-17A) T-cells, and generated non-T non-B lymphocytes that played an important role in the immune response [515]. Nevertheless, laboratory and clinical studies have been useful in identifying antigens common with the gonococcus that could be contributing to this partial protective effect. Such antigens included NHBA, the accessory proteins GNA2091 and GNA1030, PorB, TonB-dependent receptors, Rmp, PilQ, FbpA, lipoprotein NMB1126/1164, FrpB, Tbp1, OMP85, OpcA, OMP P1, LptD, MtrE, LysM, MIP, antioxidation AhpC TSA family glutaredoxin, MafA, ACP, BamA, and NspA [199,207,516–518], most definitely among many others.

Thus, emerging data appeared to show that meningococcal OMV vaccines were only a stop-gap for preventing gonorrhea. Also, it was unlikely that a singular antigen would be used to produce a true gonococcal vaccine. Thus, the consensus was that combinations of the best-performing antigens would lead, eventually, to a multi-component vaccine for preventing gonococcal infection [519].

## Conclusions

The pathogenesis of gonorrhea is a complex and not yet complete story. In this review we sought to distill the fundamental aspects of gonococcal pathogenesis and virulence, accepting that the literature was extensive and that we may have omitted some details. Accordingly, Table 2 provides the reader with a comprehensive list of previous reviews that can be studied at leisure. Understanding the biology of the gonococcus and its interactions with the host at the cellular and molecular levels will continue to unravel the pathogenesis of gonorrhea, and lead to the development of improved treatments and preventative vaccines.

**Table 2.** A comprehensive list of review papers on gonococcal pathogenesis. In this table, we provide the reader with a list of key review articles covering specific aspects of gonococcal pathogenesis in more detail. For brevity, we focus on reviews that provide molecular and cellular mechanisms underpinning gonococcal pathogenesis and include some early pioneering researchers that made significant contributions to this field, whose reviews are still worth reading. Reviews that focused on vaccines and AMR resistance were excluded. Reviews were assembled with date descending.

No.	Focus of the review	Reference
1	Sweet complexity: O-linked protein glycosylation in pathogenic *Neisseria*	[308]
2	Gonococcal PorB: a multifaceted modulator of host immune responses	[153]
3	Bacterium of one thousand and one variants: genetic diversity of *Neisseria gonorrhoeae* pathogenicity	[520]
4	Dinner date: *Neisseria gonorrhoeae* central carbon metabolism and pathogenesis	[271]
5	Mechanisms of host manipulation by *Neisseria gonorrhoeae*	[131]
6	Immune responses to *Neisseria gonorrhoeae* and implications for vaccine development	[368]
6	*Neisseria gonorrhoeae*: DNA Repair Systems and Their Role in Pathogenesis	[521]
7	Acclimation to Nutritional Immunity and Metal Intoxication Requires Zinc, Manganese, and Copper Homeostasis in the Pathogenic *Neisseriae*.	[522]
8	Molecular Regulatory Mechanisms Drive Emergent Pathogenetic Properties of *Neisseria gonorrhoeae*	[523]
9	Update on the *Neisseria* Macrophage Infectivity Potentiator-Like PPIase Protein	[518]
10	Stealthy microbes: How *Neisseria gonorrhoeae* hijacks bulwarked iron during infection	[287]
11	Experimental Urethral Infection with *Neisseria gonorrhoeae*	[501]
12	A Comprehensive Review on the Interplay between *Neisseria* spp. and Host Sphingolipid Metabolites	[524]
13	Pelvic Inflammatory Disease Due to *Neisseria gonorrhoeae* and *Chlamydia trachomatis*: Immune Evasion Mechanisms and Pathogenic Disease Pathways	[382]
14	Epithelial Haven and Autophagy Breakout in Gonococci Infection	[525]
15	Intimate Relations: Molecular and Immunologic Interactions Between *Neisseria gonorrhoeae* and HIV-1	[29]
16	Complement interactions with the pathogenic *Neisseriae*: clinical features, deficiency states, and evasion mechanisms	[526]
17	Structural Basis for Evasion of Nutritional Immunity by the Pathogenic *Neisseriae*	[286]
18	Atypical, Yet Not Infrequent, Infections with *Neisseria* Species	[427]
19	Gonorrhoea	[23]
20	Peptidoglycan O-Acetylation as a Virulence Factor: Its Effect on Lysozyme in the Innate Immune System	[527]
21	Modulation effect of vaginal mucosal microflora and susceptibility to *Neisseria gonorrhoeae* infections: a systematic review and meta-analysis	[528]
22	Biology of the Gonococcus: Disease and Pathogenesis.	[22]
23	Location, Location, Location-Commensalism, Damage and Evolution of the Pathogenic *Neisseria*	[426]
24	Human Immune Responses and the Natural History of *Neisseria gonorrhoeae* Infection.	[529]
25	Macrophage- *Neisseria gonorrhoeae* Interactions: A Better Understanding of Pathogen Mechanisms of Immunomodulation	[530]
26	Pathogenesis of *Neisseria gonorrhoeae* and the Host Defense in Ascending Infections of Human Fallopian Tube.	[398]
27	Gonococcal Defenses against Antimicrobial Activities of Neutrophils	[531]
28	*Neisseria gonorrhoeae* host adaptation and pathogenesis.	[21]
29	Subversion of nutritional immunity by the pathogenic *Neisseriae*	[285]
30	Pathogenesis of *Neisseria gonorrhoeae* in the female reproductive tract: neutrophilic host response, sustained infection, and clinical sequelae	[419]
31	The sweet side of the pathogenic *Neisseria*: the role of glycan interactions in colonisation and disease	[532]
32	The ironclad truth: how in vivo transcriptomics and in vitro mechanistic studies shape our understanding of *Neisseria gonorrhoeae* gene regulation during mucosal infection	[533]
33	Transition metals at the host-pathogen interface: how *Neisseria* exploit human metalloproteins for acquiring iron and zinc	[534]
34	Neisserial surface lipoproteins: structure, function and biogenesis	[535]
35	The genetics of *Neisseria* species	[5]
36	The transferrin-iron import system from pathogenic *Neisseria* species	[290]
37	Legless pathogens: how bacterial physiology provides the key to understanding pathogenicity	[536]
38	A bacterial siren song: intimate interactions between *Neisseria* and neutrophils.	[26]
39	Focusing homologous recombination: pilin antigenic variation in the pathogenic *Neisseria*	[537]
40	Opa proteins and CEACAMs: pathways of immune engagement for pathogenic *Neisseria*	[83]
41	Virulence determinants involved in differential host niche adaptation of *Neisseria meningitidis* and *Neisseria gonorrhoeae*	[538]
42	The role of complement in gonococcal infection of cervical epithelia	[539]
43	Contribution of interactions between complement inhibitor C4b-binding protein and pathogens to their ability to establish infection with particular emphasis on *Neisseria gonorrhoeae*	[540]
44	Species-specificity of *Neisseria gonorrhoeae* infection: do human complement regulators contribute?	[541]
45	Factor H and neisserial pathogenesis	[542]
46	Defenses against oxidative stress in *Neisseria gonorrhoeae*: a system tailored for a challenging environment	[543]
47	Protein secretion and secreted proteins in pathogenic *Neisseriaceae*	[544]
48	The molecular mechanisms used by *Neisseria gonorrhoeae* to initiate infection differ between men and women	[37]
49	Type IV pilus structure and bacterial pathogenicity	[545]
50	Mechanisms of iron acquisition by the human pathogens *Neisseria meningitidis* and *Neisseria gonorrhoeae*	[546]
51	‘Small’ talk: Opa proteins as mediators of *Neisseria*-host-cell communication	[75]
52	The role of porins in neisserial pathogenesis and immunity	[168]
53	The innate and early immune response to pathogen challenge in the female genital tract and the pivotal role of epithelial cells	[547]
54	Signal transduction pathways induced by virulence factors of *Neisseria gonorrhoeae*	[548]
55	Implications of molecular contacts and signaling initiated by *Neisseria gonorrhoeae*	[549]
56	Interactions of pathogenic *Neisseriae* with epithelial cell membranes	[117]
57	Do pathogenic *Neisseriae* need several ways to modify the host cell cytoskeleton?	[550]
58	Host cell invasion by pathogenic *Neisseriae*	[551]
59	Mechanisms of neisserial serum resistance	[552]
60	Interactions of pathogenic *Neisseria* with host cells. Is it possible to assemble the puzzle?	[553]
61	Pathogenic *Neisseriae*: complexity of pathogen-host cell interplay	[554]
62	Host cell interactions and signalling with *Neisseria gonorrhoeae*	[555]
63	The role of neisserial Opa proteins in interactions with host cells	[116]
64	Sialylation of neisserial lipopolysaccharide: a major influence on pathogenicity	[351]
65	Interaction of pathogenic *Neisseriae* with nonphagocytic cells	[556]
66	Neisserial surface variation: how and why?	[557]
67	Molecular mechanisms of antigenic variation in *Neisseria gonorrhoeae*	[558]
68	Proteases of the pathogenic *Neisseriae*: possible role in infection	[559]
69	Mechanisms of mucosal invasion by pathogenic *Neisseria*	[409]
70	The biology of the gonococcus	[560]
71	Virulence of gonococci	[561]

## Data Availability

No data were generated in this article.
